# Mitochondrial clearance and increased HSF-1 activity are coupled to promote longevity in fasted *Caenorhabditis elegans*

**DOI:** 10.1016/j.isci.2024.109834

**Published:** 2024-04-27

**Authors:** Nikolaos Tataridas-Pallas, Yahyah Aman, Rhianna Williams, Hannah Chapman, Kevin J.H. Cheng, Casandra Gomez-Paredes, Gillian P. Bates, John Labbadia

**Affiliations:** 1 Institute of Healthy Ageing, Department of Genetics, Evolution and Environment, Division of Biosciences, University College London, London WC1E 6BT, UK; 2 Huntington’s Disease Centre, Department of Neurodegenerative Disease and UK Dementia Research Institute at UCL, Queen Square Institute of Neurology, University College London, London WC1N 3BG, UK

**Keywords:** Biological sciences, Physiology, molecular biology, cell biology

## Abstract

Fasting has emerged as a potent means of preserving tissue function with age in multiple model organisms. However, our understanding of the relationship between food removal and long-term health is incomplete. Here, we demonstrate that in the nematode worm *Caenorhabditis elegans,* a single period of early-life fasting is sufficient to selectively enhance HSF-1 activity, maintain proteostasis capacity and promote longevity without compromising fecundity. These effects persist even when food is returned, and are dependent on the mitochondrial sirtuin, SIR-2.2 and the H3K27me3 demethylase, JMJD-3.1. We find that increased HSF-1 activity upon fasting is associated with elevated SIR-2.2 levels, decreased mitochondrial copy number and reduced H3K27me3 levels at the promoters of HSF-1 target genes. Furthermore, consistent with our findings in worms, HSF-1 activity is also enhanced in muscle tissue from fasted mice, suggesting that the potentiation of HSF-1 is a conserved response to food withdrawal.

## Highlights

• A single period of transient fasting enhances proteostasis and extends lifespan
• Transient fasting potentiates HSF-1 activity in worms and mice
• Enhanced HSF-1 activity following fasting is dependent on mitochondrial sirtuins
• Potentiation of HSF-1 following fasting requires the H3K27me3 demethylase JMJD-3.1

## Graphical abstract

**Figure:**
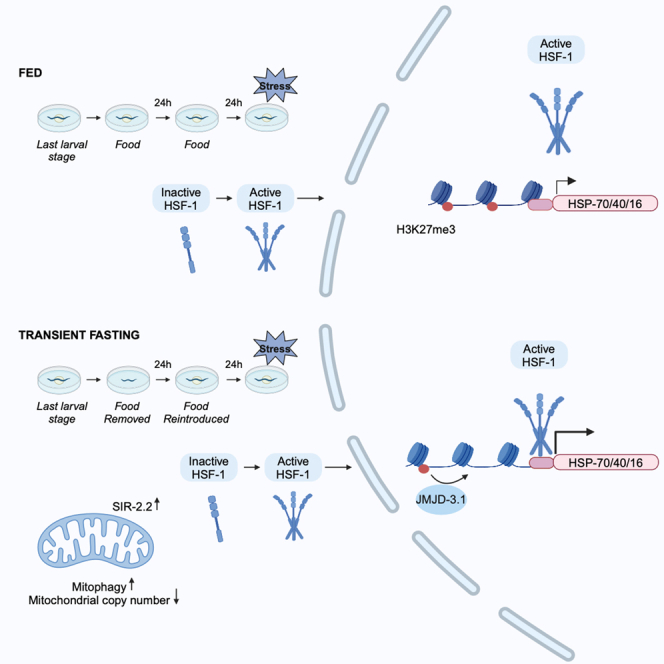

## Introduction

Maintaining a fully functional proteome is crucial for long-term cell and tissue health. This is routinely achieved by the proteostasis network (PN), a collection of molecular machines that promote accurate protein synthesis, folding, trafficking, and degradation.^1^ In parallel, the PN also rapidly recognizes and neutralizes any misfolded, mislocalized or aggregated proteins that arise from biosynthetic errors or environmental stress.^1^

As organisms age, the capacity of the PN declines, leaving tissues vulnerable to proteostasis collapse (i.e., the accumulation of misfolded, mislocalized, and aggregated proteins).^2^ This process lies at the heart of many age-associated sporadic and inherited diseases (e.g., Alzheimer’s Parkinson’s Huntington’s), raising the possibility that suppressing age-related proteostasis collapse could be a potent way to simultaneously suppress multiple age-associated diseases.^3^

Studies in the nematode worm *Caenorhabditis elegans*, have revealed that the timing and magnitude of age-related proteostasis collapse are linked to the functional status of the germline and mitochondria early in life.^4^^,^^5^^,^^6^^,^^7^ In response to impaired electron transport chain (ETC) activity or reduced levels of the mitochondrial carrier homologue, MTCH-1/MTCH2, cells can activate mitochondria-to-cytosolic stress responses that converge on the transcription factor HSF-1 to upregulate specific PN components and protect cells against proteostasis collapse later in life.^8^^,^^9^ In addition, increased HSF-1 activity has been shown to extend lifespan through mitochondria associated mechanisms,^10^^,^^11^ suggesting a close relationship between mitochondrial status, HSF-1 activity and proteome integrity. However, it remains unclear whether mitochondria-to-HSF-1 communication plays a role in promoting longevity in response to lifestyle changes that alter mitochondrial function.

Among the lifestyle interventions linked to increased lifespan, enhanced longevity as a result of altered nutrient availability is strongly linked to changes in mitochondrial status. In addition, activation of mitophagy (the primary mechanism through which defective mitochondria are removed from cells) has been shown to promote longevity and suppress age-associated disease.^12^

Nutrient restriction can take many forms, with the chronic removal or reduction of food intake through dietary restriction (DR) being one of the oldest lifestyle changes associated with increased longevity across model organisms.^13^ More recently, intermittent fasting (IF) has emerged as a more sustainable way to extend lifespan and prolong healthy tissue function.^13^ IF can take many different forms, including alternate day fasting (ADF—the removal and restoration of food on alternating days), the 5:2 diet (eating normally for 5 days and then limiting food intake to 500–600 calories for 2 days a week) and time restricted fasting (TRF—food intake is limited to short windows on each day).^14^ These lifestyle interventions are sufficient to provide long-term benefits, including extended lifespan and increased stress resistance in worms and flies.^15^^,^^16^ Furthermore, exposing worms or flies to fasting regimens exclusively during early life is sufficient to elicit lifespan extension.^15^^,^^16^

Both DR and fasting are associated with mitochondrial and metabolic remodeling,^17^^,^^18^ and HSF-1 is required for DR and TRF to increase lifespan in worms.^15^^,^^19^ This raises the possibility that the beneficial effects of fasting may be mediated by an interplay between mitochondria and HSF-1. However, it remains unknown whether mitochondria communicate with HSF-1 in response to fasting, how this is achieved, how long food removal is required for to elicit these effects and whether this can protect against age-related protein aggregation.

Here, we address these questions and demonstrate that a single period of fasting early in life potentiates HSF-1 activity, suppresses age-related protein aggregation, enhances stress resistance and extends lifespan in *C. elegans*. These effects are dependent on mitochondrial sirtuins and the H3K27me3 demethylase, JMJD-3.1, and are associated with a rapid reduction in mitochondrial copy number and a decrease in H3K27me3 levels at HSF-1 target genes.

## Results

### Early-life fasting increases proteostasis capacity in aged tissues

**Figure 1:**
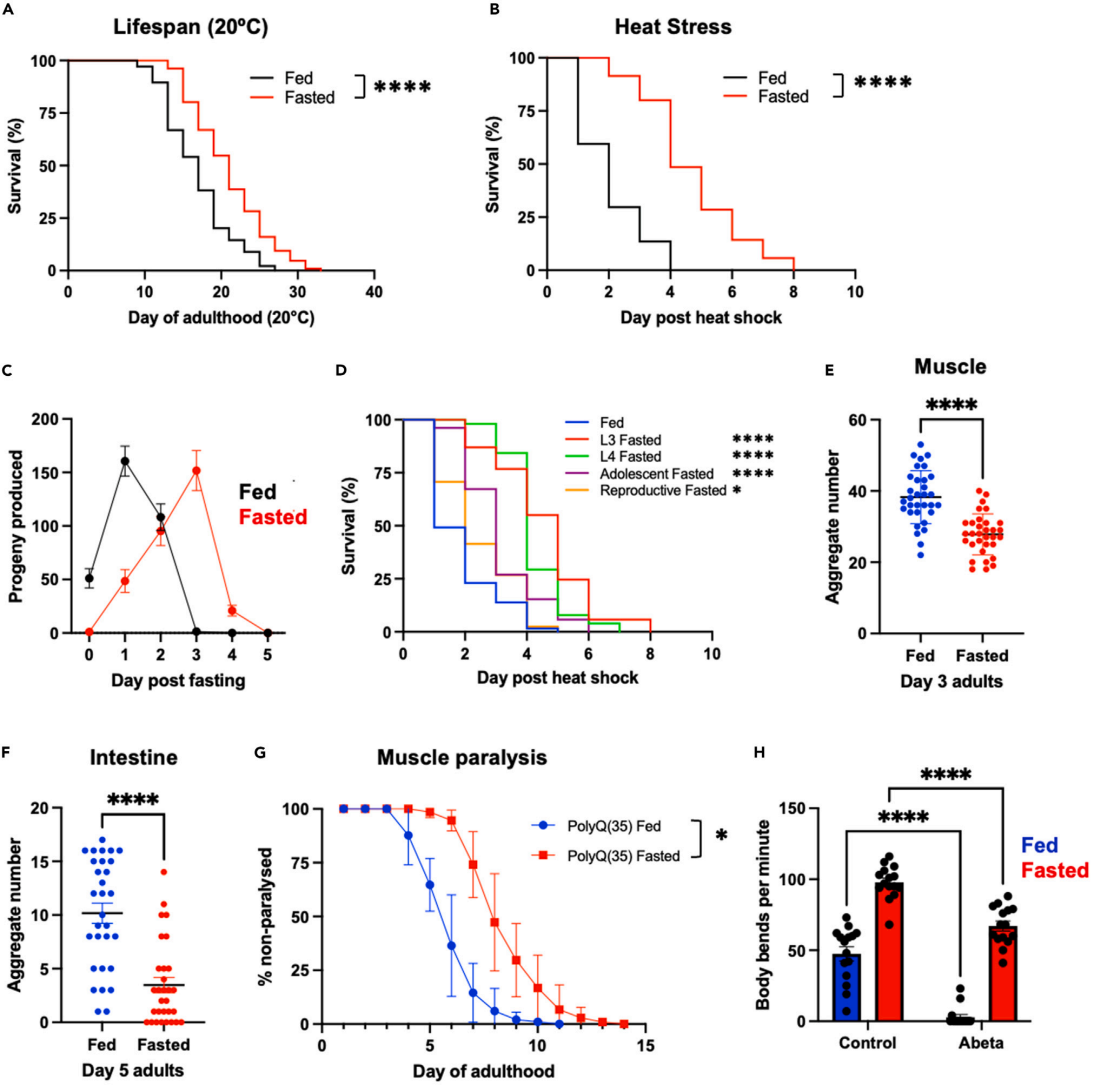
Fasting at the transition to adulthood maintains proteostasis capacity in aged tissues and extends lifespan (A and B) Survival at 20°C or following heat shock (HS) (35°C, 4 h) in fed or fasted worms. (C) Progeny produced at specific days of adulthood in fed and fasted animals. Data plotted are mean values ± SD. (D) Survival of fed or fasted animals (starting at L3, L4, day 1 of adulthood or day 2 of adulthood) following heat shock (35°C, 4 h). (E and F) PolyQ::YFP aggregate number in fed and fasted worms on day 3 and day 5 of adulthood in (E) body wall muscles and (F) intestines, respectively. Data plotted are mean values ± (E) SD or (F) SEM. (G) Proportion of motile polyQ (35) worms at different days of adulthood following constant feeding or transient fasting. Data plotted are mean values ± SD. (H) Body bends per minute (thrashing rate) of fed and fasted worms expressing ABeta in body wall muscle cells. Data plotted are mean ± SEM. Statistical comparisons were made using Mantel-Cox Log-Rank test (A & B), Student’s unpaired t-test (E and F) or two-way ANOVA (G and H). ∗ = *p* < 0.05, ∗∗∗∗ = *p* < 0.0001. Full statistics for survival curves can be found in Table S1. Fasting conditions were as follows: A, C, E, F, G, and H—animals were removed from food for 24 h starting at the L4 stage and then returned to food thereafter; B—animals were removed from food for 24 h starting at the L4 stage and then returned to food for 24 h prior to HS; C—animals were removed from food for 24 h starting at the indicated life stage and then returned to food for 24 h prior to HS. See also Figure S1 and Table S1.

TRF and 5:2 fasting have been shown to extend lifespan, prolong healthy tissue function and increase stress resistance in both worms and flies.^15^^,^^16^ In *C. elegans*, the removal of food at the transition to adulthood results in a rapid NHR-49-dependent starvation response.^17^ Given that early-life fasting is sufficient to elicit long-term health benefits, we tested whether transiently removing worms from food for 24 h as they transition to adulthood (Figure S1A), would be sufficient to extend lifespan and maintain proteostasis capacity with age. We found that worms that had been fasted for 24 h at the L4/D1 adult stage and then returned to food, exhibited a 25% increase in median and maximal lifespan (Figure 1A). In addition, fasted animals also exhibited increased resistance to heat and endoplasmic reticulum (ER) stress (Tunicamycin treatment), but less so to oxidative stress (paraquat treatment), when returned to food for 24 h and allowed to commence reproduction (Figures 1B, S1B, and S1C). These effects were not associated with reduced brood size, although the onset of egg-laying was delayed in fasted animals (Figures 1C and S1D). However, a 24-h period of fasting was required before the onset of reproductive maturity for maximal enhancement of stress resistance (Figure 1D). These data demonstrate that a single, 24 h fast, prior to the onset of reproduction, is sufficient to increase lifespan and stress resistance in *C. elegans*, even when animals are returned to food.

Increased stress resistance in reproductively mature worms has previously been linked to the maintenance of proteostasis capacity in adulthood and the suppression of age-related protein aggregation.^4^^,^^5^ To ascertain whether a single period of early-life fasting maintains proteostasis capacity with age, we examined the effect of transient food removal on polyglutamine (polyQ) aggregation and toxicity in body wall muscle and intestinal cells. Consistent with increased lifespan and stress resistance, we found that fasting suppressed age-related polyQ aggregation in both tissues (Figures 1E and 1F) and delayed the onset of muscle paralysis caused by polyQ proteins (Figure 1G). In addition, fasting also improved muscle function in control and Abeta1-42 expressing worms (Figure 1H).

Moreover, fasting further increased stress resistance in germline stem cell (GSC) deficient, chronically dietary restricted (DR) and ETC compromised mutants (Figures S1E–S1G), suggesting that fasting may enhance proteostasis capacity through different mechanisms than these interventions. Our data demonstrate that a single, transient period of fasting early in life is sufficient to suppress age-related proteostasis collapse in different tissues and promote longevity without compromising reproduction.

### Fasting enhances stress resistance and increases lifespan by potentiating HSF-1 activity and selectively remodeling the proteostasis network

Proteostasis capacity is controlled through the activity of well-established transcription factors that balance the expression of PN components (e.g., molecular chaperones and degradation factors) with the protein folding requirements of the cell.^1^^,^^2^ To determine which, if any, of these PN regulators are required for fasting to enhance proteostasis capacity, we examined resistance to heat stress in worms defective in the HSR (*hsf-1(sy441)*, *daf-16(mu86)*, *pqm-1(ok485)*), UPR^ER^ (*xbp-1(zc12)*, *atf-6(tm1153)*, *pek-1(ok275)* and *ire-1(ok799)*) and UPR^mt^ (*atfs-1(tm4525)*).

**Figure 2:**
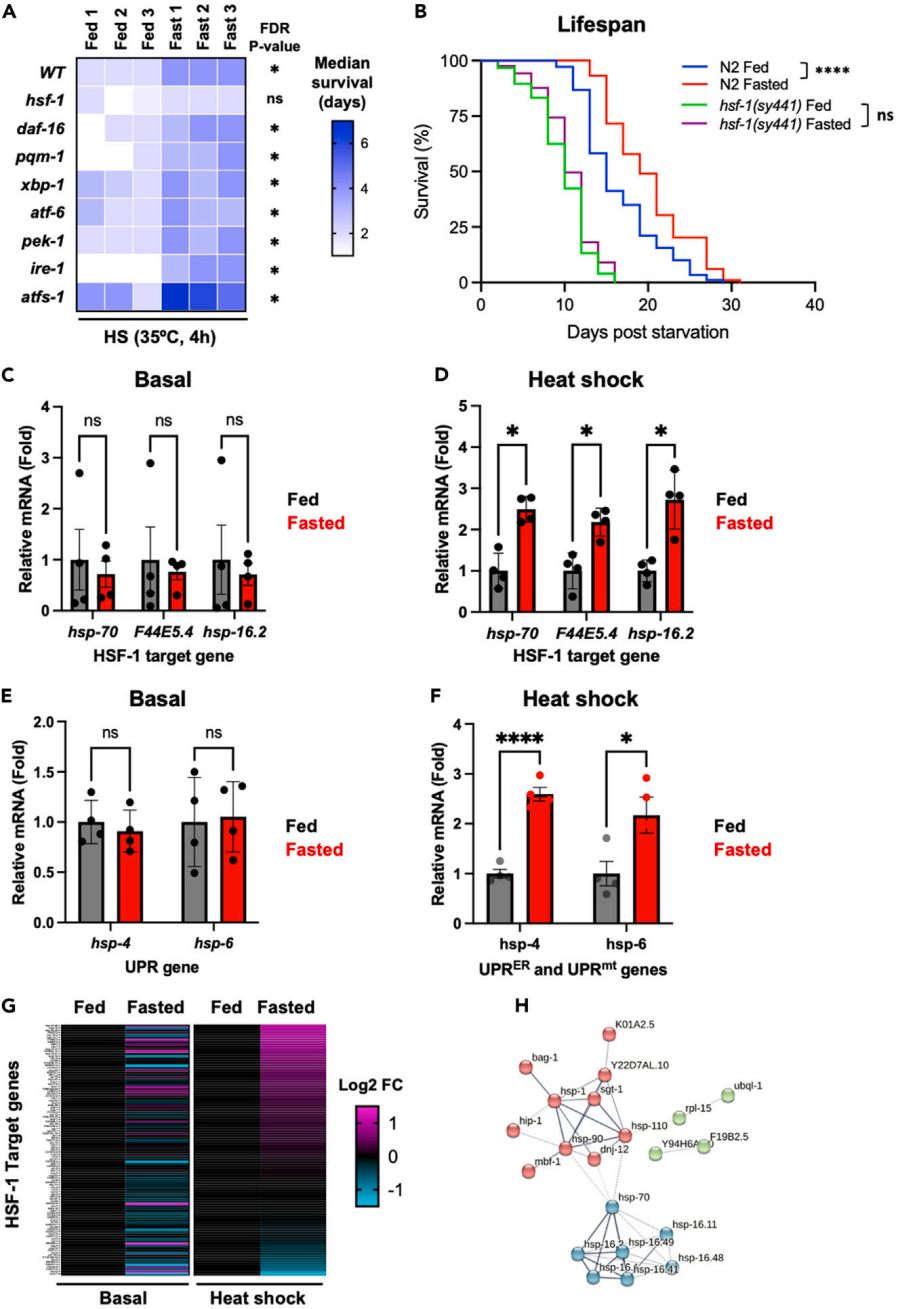
Fasting enhances proteostasis capacity and promotes longevity through the selective potentiation of HSF-1 activity (A) Survival of fed and fasted wild-type (N2) or PN mutant animals following heat shock (HS) (35°C, 4 h). (B) Survival at 20°C of fed and fasted wild-type and *hsf-1(sy441)* mutant worms. Lifespan assay was run in parallel with those presented in Figures 3G and 3H. (C and D) Relative mRNA levels of canonical HSF-1 target genes in fed and fasted worms under (C) basal (20°C) and (D) HS (35°C, 30 min) conditions. Data plotted are mean ± (C) SEM or (D) SD. (E and F) Relative mRNA levels of *hsp-4* and *hsp-6* in fed and fasted worms under (E) basal or (F) HS (35°C, 30 min) conditions. Data plotted are mean ± (E) SD or (F) SEM. (G) Relative expression of all HSF-1 target genes in fed and fasted worms under basal or HS (35°C, 30 min) conditions. (H) STRING network of up-regulated (*p* < 0.05) HSF-1 target PN genes in fasted worms following HS (35°C, 30 min). For panels C–G, worms were harvested immediately after the heat shock conditions specified. Statistical comparisons were made using Mantel-Cox Log-Rank test (B) or Student’s unpaired t-test with FDR correction (C–F). ns = *p* > 0.05, ∗ = *p* < 0.05, ∗∗∗∗ = *p* < 0.0001. Full statistics for heatmap and survival curves can be found in Table S1. Fasting conditions were as follows: A, C, D, E, and F—animals were removed from food for 24 h starting at the L4 stage and then returned to food for 24 h prior to HS; B—animals were removed from food for 24 h starting at the L4 stage and then returned to food thereafter; G—animals were removed from food for 24 h starting at the L4 stage and then immediately exposed to basal or HS conditions. See also Figure S2, Tables S1 and S4.

As expected, wild-type worms exposed to fasting exhibited a 2-fold increase in median survival following heat stress, as compared to fed controls (Figure 2A). Although we cannot rule out the presence of adaptive changes to parallel PN pathways in our strains, mutations in *daf-16*, *pqn-1*, *xbp-1*, *atf-6*, *pek-1* or *ire-1* did not block the ability of fasting to enhance stress resistance (Figures 2A and S2B–S2G), while survival was markedly increased in *atfs-1(tm4525)* mutants (Figures 2A and S2H). In contrast, elevated stress resistance was almost completely abolished in fasted *hsf-1(sy441)* loss-of-function mutants (Figures 2A and S2A). Consistent with this, loss of HSF-1 activity also prevented fasting from increasing lifespan (Figure 2B).

To determine whether HSF-1 activity is increased in transiently fasted animals, we measured the basal and heat-induced expression of canonical HSF-1 target genes (*hsp-70*, *F44E5.4*, *hsp-16.2*) in worms that had been fasted for 24 h at the L4 stage and allowed to recover for 24 h in the presence of food. The basal expression of HSF-1 target genes, or canonical UPR^ER^ or UPR^mt^ genes, was not increased under basal conditions (Figures 2C and 2E). However, the induction of canonical genes from all three pathways was increased 2- to 3-fold in transiently fasted worms immediately following heat shock (Figures 2D and 2F). These data suggest that transient fasting simultaneously potentiates proteostastic stress responses associated with the cytosol/nucleus, ER and mitochondria.

Using RNA-sequencing, we asked whether fasting broadly or selectively enhanced HSF-1 activity. Worms were fasted for 24 h at the L4 stage and then collected immediately following exposure to control (basal) conditions (20°C) or heat shock (35°C) for 30 min. Of the 107 genes known to be directly regulated by HSF-1 during development or in response to heat shock,^20^ only a small sub-set (9 significantly upregulated and 9 significantly downregulated) were found to be significantly changed in fasted animals compared to fed animals, under basal conditions (*p* < 0.05) (Figure 2G). In contrast, the expression of a far larger proportion of HSF-1 targets (31 significantly uregulated and 2 significantly downregulated) was significantly altered in fasted animals compared to constantly fed controls, following heat shock (*p* < 0.05) (Figure 2G). Of these genes, only 5 were concordantly up- (*F19B2.5*, *cdd-1*, *Y94H6A.10*, *Y22D7AL.10*) or downregulated (*egg-5*) in fasted animals compared to fed controls under both basal and heat shock conditions (Figure 2G).

Of the HSF-1 target genes that exhibited increased expression following heat shock in fasted animals compared to fed controls, many encoded for core components of key proteostasis machines, including the HSP70/DNAJ machinery (*hsp-70*, *dnj-12*, *bag-1*), the disaggregase machinery (*hsp-1*, *hsp-110*, *dnj-12*), non ATP-dependent chaperones (*hsp-16.1*, *hsp-16.11*, *hsp-16.2*, *hsp-16.41*, *hsp-16.48*, *hsp-16.49*), and the HSC70/HSP90 complex (*hsp-1*, *hsp-90*, *hip-1*, *sgt-1*) (Figure 2H). We did not see substantial changes in the expression of proteasomal subunits or components of the autophagy machinery. Moreover, fluorescence-based reporters of proteasome activity^21^ or autophagic flux^22^ revealed that neither of these pathways were enhanced in worms that had been transiently fasted, compared to constantly fed controls (Figures S2I–S2O). In fact, degradation of a UbV::GFP proteasomal substrate was reduced in fasted animals under both basal and heat shock conditions (Figures S2M–S2O).

Together, our data show that *hsf-1* is required for fasting to promote longevity and enhance proteostasis capacity, and that these beneficial effects are associated with the increased expression of a sub-set of HSF-1 target genes, either basally, or in response to stress. While central regulators of the UPR^ER^ and UPR^mt^ were not required for increased resistance to heat stress (Figure 2A), it remains possible that the potentiation of these pathways, and perhaps other PN factors/pathways, also contributes to resistance to other stresses and/or increased longevity.

### Fasting induced stress resistance is dependent on mitochondrial sirtuins and mitophagy

**Figure 3:**
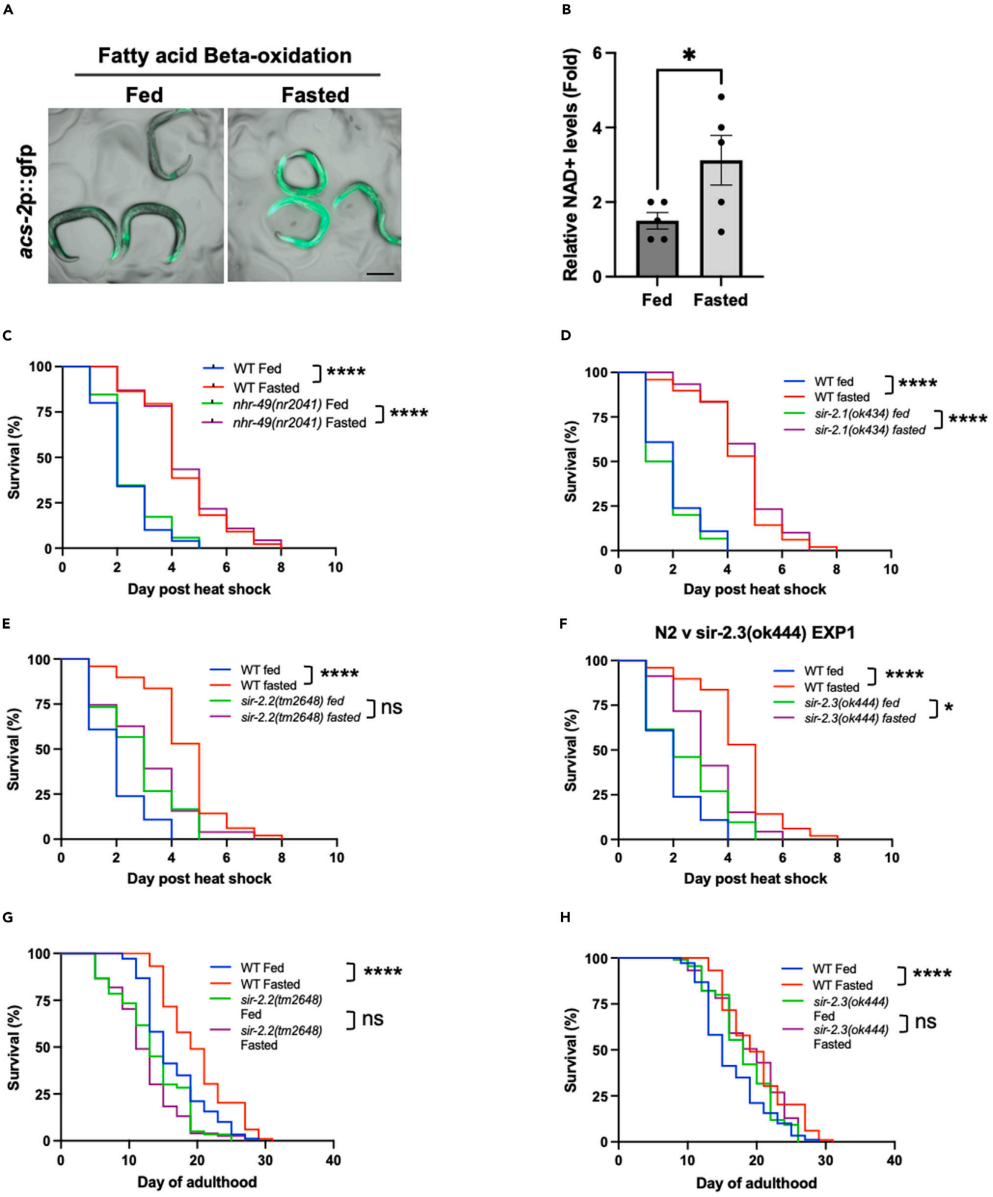
Mitochondrial sirtuins are necessary for increased stress resistance and longevity following early-life fasting (A) Representative images of fed and fasted WBM321 acs-2p:gfp worms. Scale bar, 200 μM. (B) Relative NAD+ levels in fed and fasted worms. Data plotted are mean ± SEM. (C–F) Survival of fed and fasted wild-type (N2), (C) *nhr-49(nr2041)*, (D) *sir-2.1(ok434)*, (E) *sir-2.2(tm2648)* and (F) *sir-2.3(ok444)* mutant worms following heat shock (35°C, 4 h). (G and H) Lifespan at 20°C of fed and fasted wild-type (N2), (G) *sir-2.2(tm2648)* and (H) *sir-2.3(ok444)* mutants. Statistical comparisons were made using Student’s unpaired t-test (B) and Mantel-Cox Log-Rank test (C–H). ns = *p* > 0.05, ∗ = *p* < 0.05, ∗∗∗∗ = *p* < 0.0001. Full statistics for survival curves can be found in Table S1. Survival assays in D–F, and G and H (also run alongside survival assay presented in Figure 2B), were run in parallel. Fasting conditions were as follows: A–F—animals were removed from food for 24 h starting at the L4 stage and then returned to food for 24 h prior to imaging, collection or HS; G and H—animals were removed from food for 24 h starting at the L4 stage and then returned to food thereafter. See also Figure S3 and Table S1.

Fasting is associated with increased fatty acid beta oxidation, altered mitochondrial morphology and elevated NAD+ levels in worms and mice.^17^^,^^23^^,^^24^ Consistent with previous reports, we observed increased *acs-2*p::gfp expression (a hallmark of increased beta-oxidation), increased NAD+ levels and a more fragmented mitochondrial network in worms that had been fasted for 24 h and then allowed to recover for 24 h in the presence of food (Figures 3A, 3B, S3A, and S3B). In addition, RNA-seq data from fasted animals exhibited a strong enrichment for metabolic pathways among differentially expressed genes (1,667 upregulated, 999 downregulated, *p* < 0.05) (Figures S3C and S3D).

Metabolic and mitochondrial remodeling in response to nutrient deprivation are dependent on the transcription factor NHR-49, and the lysine deacetylase SIR-2.1 (whose activity is increased in response to elevated NAD+ levels).^17^^,^^23^ Given that both NHR-49 and SIR-2.1 have been linked to HSF-1 activity,^11^^,^^25^^,^^26^ we hypothesized that one or both of these factors may be necessary for fasting induced stress resistance and longevity.

To address this, we measured stress resistance in fed and transiently fasted (24 h fasting followed by 24 h recovery on food) loss-of-function *nhr-49(nr2041)* and *sir-2.1(ok434)* mutants. Surprisingly, *nhr-49* and *sir-2.1* mutants exhibited comparable stress resistance to wild-type worms under both fed and fasted conditions (Figures 3C and 3D). Given that we observed increased NAD+ levels and altered mitochondrial homeostasis in fasted animals, we also tested whether the mitochondrial sirtuins, SIR-2.2 and SIR-2.3,^27^ are required for fasting to increase stress resistance. We observed that stress resistance was mildly increased in *sir-2.2(tm2648)* and *sir-2.3(ok444)* mutants under fed conditions, although not to the same extent as is observed in fasted wild-type animals (Figures 3E and 3F). Crucially, fasting failed to increase stress resistance or lifespan in *sir-2.2* or *sir-2.3* mutants (Figures 3E–3H), although it should be noted that loss of *sir-2.3* increased lifespan under fed conditions to a level comparable with that seen following fasting (Figure 3H). Together, our data suggest that SIR-2.2 and SIR-2.3 are required for fasting to fully enhance stress resistance and increase longevity.

### SIR-2.2 couples reduced mitochondrial copy number with increased HSF-1 activity to promote longevity

**Figure 4:**
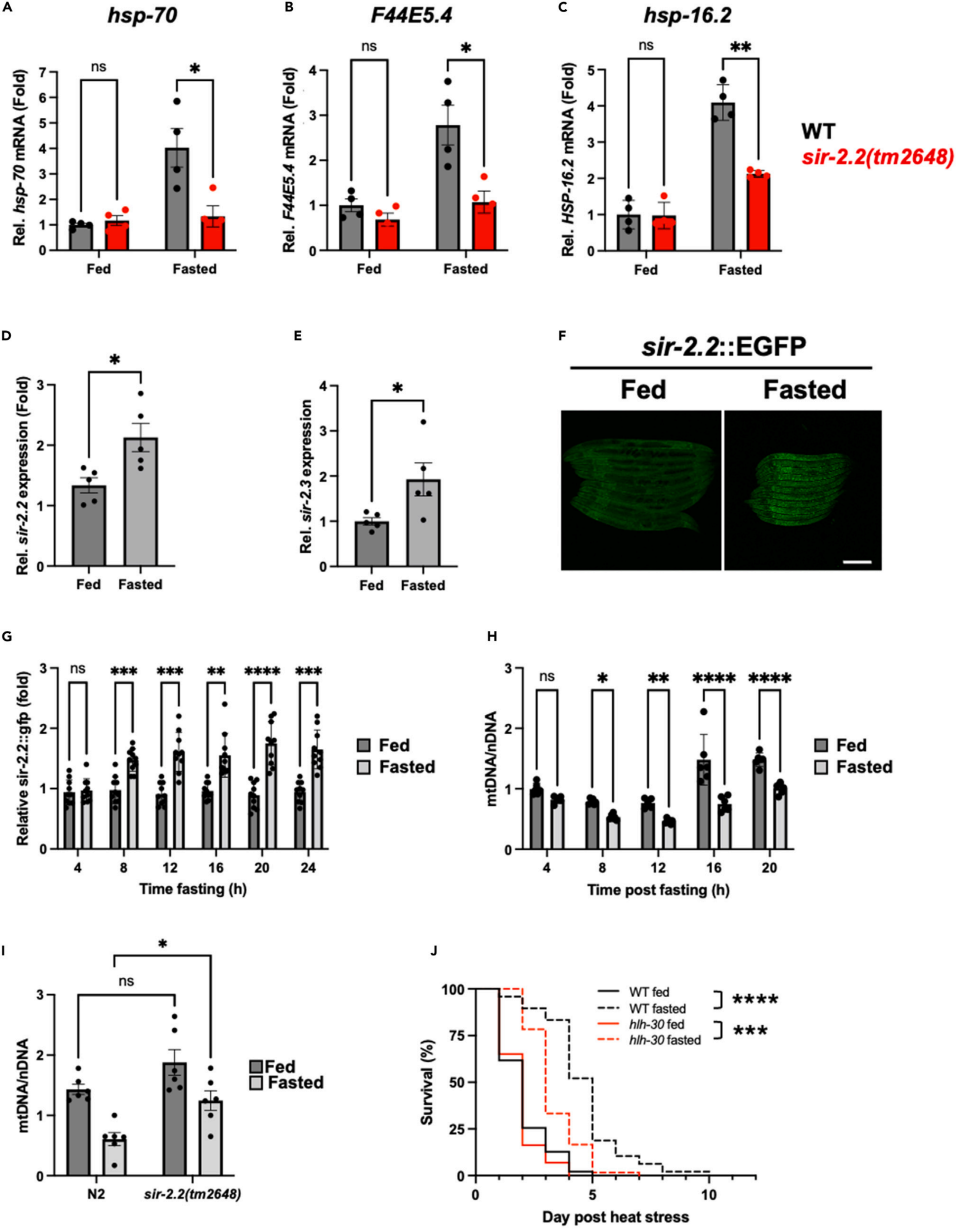
SIR-2.2 couples mitochondrial clearance with enhanced HSF-1 activity in fasted animals (A–C) Relative expression of HSF-1 target genes in fed and fasted wild-type worms or *sir-2.2(tm2648)* mutants following heat shock (35°C, 30 min). Worms were harvested immediately following heat shock. Data are plotted as mean ± (A and B) SEM or (C) SD. (D and E) Relative *sir-2.2* and *sir-2.3* mRNA levels in fed and fasted animals. Data are plotted as mean ± SEM. (F) Representative images of fed and fasted SIR-2.2:EGFP worms. Scale bar, 200 μM. (G & H) Relative (G) SIR-2.2:EGFP levels and (H) mitochondrial copy number at different times post fasting. Data are plotted as mean ± SD. (I) Mitochondrial copy number in fed and fasted wild-type or *sir-2.2(tm2648)* mutant worms. Data are plotted as mean ± SEM. (J) Survival of fed and fasted wild-type (N2) and *hlh-30* mutant worms following heat shock (35°C, 4 h). Statistical comparisons were made using two-way ANOVA with post-analysis pairwise comparison of groups (A–C and G–I), Student’s unpaired t-test (D and E) and Mantel-Cox Log-Rank test (J). ns = *p* > 0.05, ∗ = *p* < 0.05, ∗∗ = *p* < 0.01, ∗∗∗ = *p* < 0.001, ∗∗∗∗ = *p* < 0.0001. Full statistics for survival curves can be found in Table S1. The survival assay in J was run in parallel with S4C and D. Fasting conditions were as follows: A–C and J—animals were removed from food for 24 h starting at the L4 stage and then returned to food for 24 h prior to HS; D–F—animals were removed from food for 24 h starting at the L4 stage and then immediately collected or imaged; G and H—animals were removed from food at the L4 stage for the indicated times before collection; I—animals were removed from food at the L4 stage for 16 h before collection. See also Figure S4, Tables S1 and S4.

To understand the relationship between fasting, mitochondrial sirtuins, HSF-1 activity and maintenance of proteostasis, we first asked whether *sir-2.2* was required for potentiation of HSF-1 activity. As expected, *hsp-70*, *F44E5.4*, and *hsp-16.2* mRNA were increased 3- to 4-fold in transiently fasted animals (24 h fasting followed by 24 h recovery on food) immediately following heat shock (Figures 4A–4C). In contrast, this response was severely blunted in *sir-2.2* mutants (Figures 4A–4C), suggesting that *sir-2.2* is required for fasting to increase HSF-1 activity in response to stress.

To determine whether SIR-2.2 or SIR-2.3 is elevated in response to fasting, we measured *sir*-2.2 and *sir-2.3* mRNA levels in fed and fasted animals. Both *sir-2.2* and *sir-2.3* mRNA were increased 1.5-fold and 2-fold, respectively, following 24 h of fasting (no recovery on food) (Figures 4D and 4E). In addition, we observed increased fluorescence in fasted worms expressing SIR-2.2:GFP^28^ (Figure 4F), indicating that SIR-2.2 levels, and presumably activity, are increased by fasting.

Given that SIR-2.2 is a mitochondrial protein,^28^ we hypothesized that increased SIR-2.2:GFP levels may be reflective of increased mitochondrial biogenesis. To test this, we measured SIR-2.2:GFP levels and mitochondrial copy number every 4 h following removal of food. SIR-2.2:GFP levels increased approximately 1.5-fold after 8 h of fasting and persisted at this level thereafter (Figure 4G). In contrast with our hypothesis, this was coincident with a 30–40% decrease in mitochondrial copy number within the same time window (Figure 4H). These effects were also observed in germline-less *glp-1(e2144ts)* mutants (Figures S4A and S4B), indicating that mitochondrial copy number is rapidly reduced within the soma of fasted worms. Furthermore, this reduction in mitochondrial copy number was suppressed in fasted *sir-2.2* mutants compared to wild-type animals (Figure 4I), suggesting that elevated levels of SIR-2.2 may promote mitochondrial turnover in response to fasting.

Given that imbalances in copy number between the mitochondrial and nuclear genomes can result in mitochondrial stress responses, and that *sir-2.2* is necessary for the potentiation of HSF-1, we reasoned that mitochondrial clearance may be coupled with HSF-1 activity to promote proteostasis capacity. Mitophagy is the primary mechanism by which mitochondria are removed from cells if UPS-based repair is not sufficient to correct mitochondrial defects^12^^,^^29^; to test whether mitochondrial clearance was required for potentiation of HSF-1 activity, we measured stress resistance in worms with impaired lysosomal biogenesis (*hlh-30(tm1978)*) or defective PINK1 and parkin (*pink-1(tm1779)* and *pdr-1(gk448)*), all of which are required for efficient mitophagy.^30^^,^^31^

Consistent with a coupling of mitochondrial clearance and increased HSF-1 activity, we found that stress resistance was reduced (but not abolished) in *hlh-30*, *pink-1*, and *pdr-1* mutant worms following transient fasting (Figures 4J, S4C, and S4D), suggesting that mitophagy is required for transient food withdrawal to enhance organismal robustness. However, the fact that mitophagy defective mutants remained more stress resistant than fed counterparts suggests that pathways beyond mitophagy may also contribute to stress resistance following temporary food withdrawal.

Together, our data support a model in which elevated levels of SIR-2.2 potentiate HSF-1 activity through mitochondrial clearance in fasted animals, thereby enhancing proteostasis capacity and promoting longevity. However, it remains possible that SIR-2.2 may also control mitochondrial copy number by influencing mechanisms outside of mitophagy, such as mitochondrial biogenesis.

### JMJD-3.1 promotes HSF-1 activity in response to fasting

How then, do elevated SIR-2.2 levels and reduced mitochondrial copy number influence HSF-1 activity in the nucleus? Given that changes in mitochondrial import drive mitophagy,^29^ we reasoned that SIR-2.2 may translocate to the nucleus in response to fasting. However, we did not observe any evidence for the nuclear localization of SIR-2.2:GFP in fasted animals (Figure S5A).

We next considered how SIR-2.2 could remotely influence HSF-1 activity. Mitochondrial stress responses and homeostasis are dependent on the H3K27me3 demethylase JMJD-3.1 and the lysine acetyltransferase CBP-1.^32^^,^^33^ In addition, increased expression of *jmjd-3.1* promotes HSF-1 activity following GSC removal in worms.^5^ Therefore, we hypothesized that SIR-2.2 dependent changes in mitochondrial copy number might potentiate HSF-1 activity by increasing JMJD-3.1 activity.

**Figure 5:**
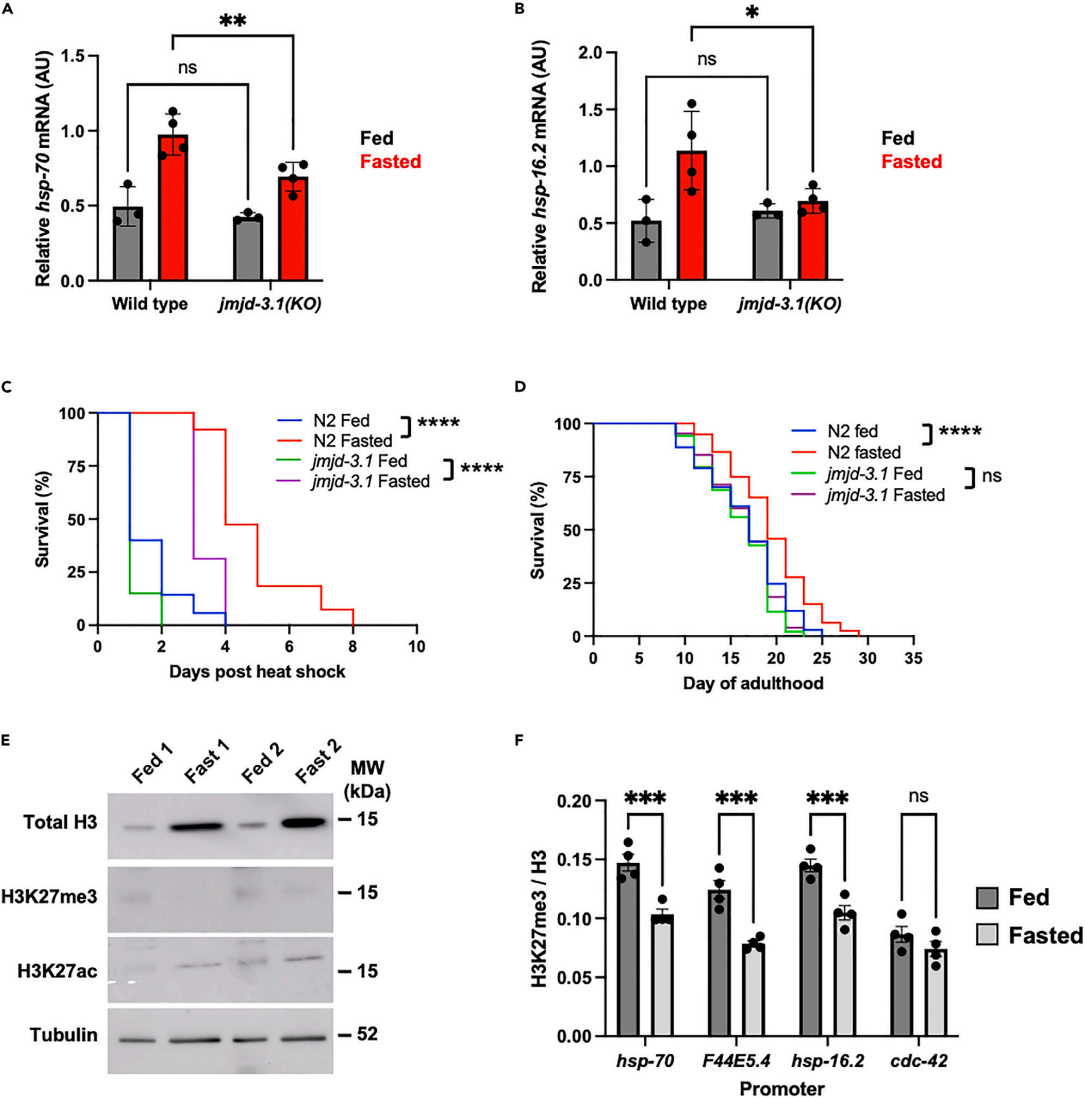
JMJD-3.1 promotes HSF-1 potentiation in response to fasting (A and B) Relative mRNA (arbitrary units, AU) of HSF-1 target genes in heat shocked (35°C, 30 min) wild-type and *jmjd-3.1(gk384) KO* mutant worms. Worms were harvested immediately following heat shock. Data are plotted as mean ± SD. (C) Survival of fed and fasted wild-type and *jmjd-3.1(gk384)* mutant worms following heat shock (35°C, 4 h). (D) Lifespan at 20°C of wild-type and *jmjd-3.1(gk384)* mutant worms. (E) Representative western blots of total histone H3, H3K27me3, H3K27ac, and tubulin in fed and fasted worms. (F) Relative H3K27me3 levels at HSF-1 target promoters in fed and fasted worms. Data are plotted as mean ± SEM. Statistical comparisons were made using two-way ANOVA with post-analysis pairwise comparison of groups (A and B), Mantel-Cox Log-Rank test (C and D) and unpaired Student’s t test (F). ns = *p* > 0.05, ∗ = *p* < 0.05, ∗∗∗ = *p* < 0.001, ∗∗∗∗ = *p* < 0.0001. Full statistics for survival curves can be found in Table S1. Fasting conditions were as follows: A–C and F—animals were removed from food for 24 h starting at the L4 stage and then returned to food for 24 h prior to HS or collection; D—animals were removed from food for 24 h starting at the L4 stage and then returned to food thereafter; E—animals were removed from food for 24 h starting at the L4 stage and then immediately collected. See also Figure S5, Tables S1 and S4. (see also Figure S5).

To test this, we exposed wild type and *jmjd-3.1* knock out (KO) worms to transient fasting (24 h fasting followed by 24 h recovery on food) and then measured the expression of HSF-1 target genes immediately following heat shock (35°C, 30 min). We observed that transiently fasted *jmjd-3.1* mutants were unable to increase the expression of HSF-1 target genes in response to heat shock to the level observed in transiently fasted wild type animals (Figures 5A and 5B). In addition, transiently fasted *jmjd-3.1* KO mutants were also not as stress resistance or long-lived as transiently fasted wild-type animals (Figures 5C and 5D). Similarly, we found that knockdown of *cbp-1* also suppressed stress resistance in fasted animals compared to empty vector (L4440) controls (Figure S5B). Consistent with an increase in JMJD-3.1 activity, we also found that total H3K27me3 levels were strongly reduced in transiently fasted animals compared to constantly fed counterparts (Figure 5E) and that H3K27me3 levels were reduced in transiently fasted worms by 20–40% at the promoters of *hsp-70*, *F44E5.4*, and *hsp-16.2*, but not at the promoter of *cdc-42*, whose expression is not regulated by HSF-1 or altered by fasting (Figure 5F). Surprisingly, reduced levels of H3K27me3 did not correlate with an increase in total levels of H3K27ac and were not due to a reduction in histone H3, the levels of which were highly elevated (Figure 5E), suggesting a substantial change in chromatin state occurs in response to fasting.

Together, our data suggest that in response to reduced mitochondrial copy number, JMJD-3.1 promotes HSF-1 activity in response to fasting through the demethylation of histone H3 at the promoters of HSF-1 target genes.

### Fasting selectively potentiates HSF-1 activity in mouse skeletal muscle but not brain tissue

**Figure 6:**
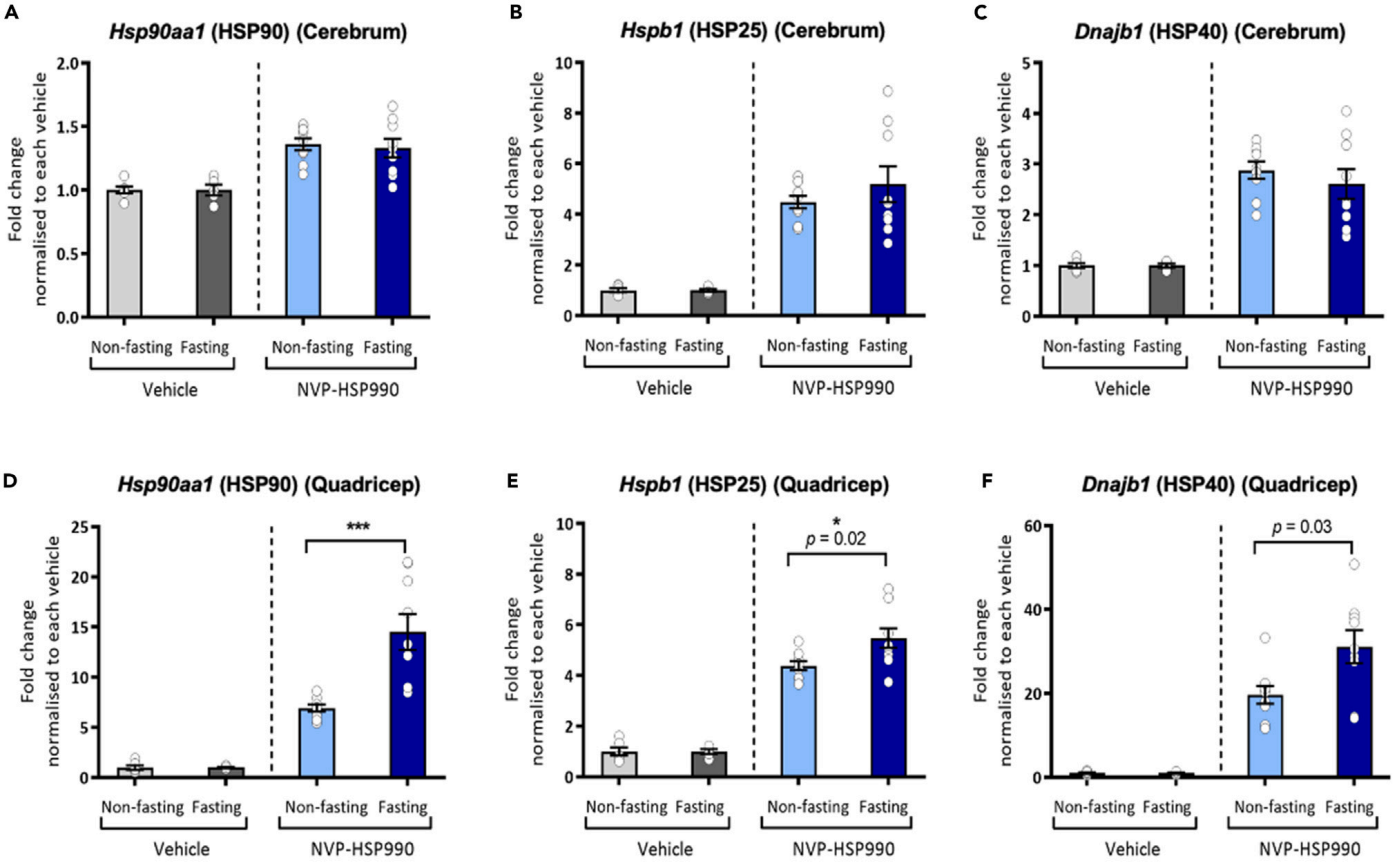
Fasting selectively potentiates HSF-1 activity in mouse skeletal muscle but not brain tissue (A–C) QuantiGene assessment of HSF-1 target genes in brain tissue of fed and fasted mice following treatment with vehicle or NVP-HSP990 (12 mg/kg). Values following NVP-HSP990 treatment were normalized to corresponding vehicle controls. Data plotted are mean ± SEM. (D–F) QuantiGene assessment of HSF-1 target genes in quadricep muscle tissue of fed and fasted mice following treatment with vehicle or NVP-HSP990 (12 mg/kg). Data plotted are mean ± SEM. Statistical comparisons were made using two-way ANOVA with Bonferroni correction. ∗∗∗*p* < 0.001. Data were screened for outliers using a ROUT test and one mouse was removed from both the NVP-HSP990 fed and fasted groups. Final sample numbers were: vehicle non-fasting = 4, vehicle fasted = 6, NVP-HSP990 non-fasting = 9, NVP-HSP990 fasted = 9. See also Figure S6, Tables S2–S4. (see also Figure S6).

Various forms of fasting are associated with lifespan extension in worms, flies, and mammals.^14^ Therefore, we tested whether fasting also increases HSF-1 activity in mice using a brain permeable HSF1 activator (NVP-HSP990).^34^ We found that mice subjected to fasting for 16 h exhibited no increase in the basal expression of HSF1 target genes in quadricep muscle or brain tissue (cerebrum), with *Hspa1a/b*, *Hspb1*, *Dnajb1*, and *Hsph1* mRNA decreasing in the cerebrum (Figures S6A and S6B). Similarly, fasting had no effect on the fold induction of HSF1 target genes in the cerebrum following treatment with NVP-HSP990 (Figures 6A–6C and S6C–S6E). In contrast, the induction of *Hsp90aa1*, *Hspb1*, and *Dnajb1* following NVP-HSP990 treatment, was increased in the quadriceps of fasted mice compared to fed controls (Figures 6D–6F). However, this was not seen for *Hspa1a/b*, *Dnaja1*, or *Hsph1* (Figures S6F–S6H). Together, our data demonstrate that fasting selectively potentiates HSF1 activity in mammals, but that these effects occur in a tissue-specific manner.

## Discussion

Here, we have shown that a single, transient period of fasting, early in life, is sufficient to enhance organismal robustness and extend lifespan. These effects were associated with an enhanced ability to suppress polyglutamine aggregation in different tissues, suggesting that fasting is an effective way to maintain a healthy proteome. This is supported by earlier indications that fasting prevents the accumulation of carbonylated proteins in aged worms.^35^

Previous fasting regimens have either continuously removed food or exposed animals to multiple rounds of fasting and re-feeding in adulthood to extend lifespan.^14^ Surprisingly, we find that if food removal is administered prior to reproductive maturity, a single, transient period of fasting is sufficient to elicit beneficial effects without compromising fecundity. This suggests that as well as the duration/pattern of fasting, the stage of life when fasting is administered is a key determinant of longevity and health outcomes. However, it should also be noted that chronic or repeated removal of food can still increase lifespan, even when initiated later in adulthood.^15^^,^^36^

In *C. elegans*, the transition to reproductive maturity is marked by the repression or remodeling of numerous proteostasis pathways, including the heat shock response.^5^^,^^37^^,^^38^ Removing germline stem cells (GSCs) or compromising egg-shell integrity prevents repression of the HSR, maintains stress resistance and protects animals against age-related proteostasis collapse.^4^^,^^7^ Chronic fasting has been linked to defects in reproduction^39^; however, the fasting conditions employed here did not lead to a reduction in total brood size, suggesting that the effects we observe arise independently of changes to the germline. The fact that an analogous response is also present in mouse muscle tissues further argues that the potentiation of HSF-1 activity is a general cellular response to fasting.

An important unanswered question is, how exactly does fasting promote longevity and tissue health? Increased lifespan in response to fasting has been linked to altered metabolism, mitochondrial remodeling and increased activity of PN pathways, including the ubiquitin proteasome system, the UPR^ER^ and autophagy.^35^^,^^40^^,^^41^ We find that in addition to these pathways, the potentiation of HSF-1 activity in response to protein folding stress is also a key factor in fasting induced longevity. Consistent with this, HSF-1 is also necessary for lifespan extension in response to altered nutrient signaling,^15^^,^^19^^,^^42^^,^^43^ suggesting that HSF-1 is broadly required for the coupling of nutrient signaling and lifespan.

The relationship between early-life fasting and HSF-1 appears to be complex, with the expression of a distinct sub-set of HSF-1 target genes (sHSPs, HSP70s, DNAJs, and NEFs) being increased in response to protein folding stress but not increased under basal conditions. In fact, our data in mice suggests that the basal expression of some HSF-1 target genes declines in the brain under fasted conditions, an observation that parallels those previously reported in the livers of fasted mice and that has been linked to increased PGC1alpha activity.^44^ In addition, HSF-1 activity was potentiated within the muscles but not the brain tissues of fasted mice, possibly due to the increased resistance of the brain to fasting as compared to other organs.^45^ However, we cannot exclude the possibility that the responses identified in muscle cells are not present in neurons.

Increased HSF-1 activity has recently emerged as a key component of the cellular response to mitochondrial disturbances.^6^^,^^46^^,^^47^^,^^48^ Our data suggest that the potentiation of HSF-1 activity and the preservation of proteostasis capacity in fasted animals arises from altered mitochondrial homeostasis, specifically, elevated levels of mitochondrial sirtuins and reduced mitochondrial copy number within the first 8 h of fasting. Mitophagy is the primary route by which mitochondria are removed from cells and is responsive to increased NAD+ levels and lysosomal biogenesis.^30^^,^^49^^,^^50^ Furthermore, increased mitophagy has been shown to be necessary for lifespan extension in response to DR, while reduced mitophagy is associated with shortened lifespan.^30^^,^^49^

We find that NAD+ levels are elevated in fasted worms, and that HLH-30 (the master regulator of lysosomal biogenesis genes), and mitophagy adapters are necessary for full stress resistance in fasted animals. HLH-30 is required for increased lifespan in response to altered mitochondrial dynamics and chronic DR,^31^^,^^51^ and increased neuronal HLH-30 promotes heat stress resistance through mitochondrial fragmentation in muscle tissues.^52^ Furthermore, interactions between HLH-30 and HSF-1 have been proposed to govern autophagy, metabolism, and stress resistance.^53^^,^^54^ Therefore, we propose that in response to fasting, HLH-30 promotes organismal robustness through a combination of macroautophagy and mitophagy, which then stimulates HSF-1 activity to further enhance protein folding and degradation.

Our finding that elevated levels of SIR-2.2 and SIR-2.3 are necessary for mitochondrial clearance and enhanced HSF-1 activity in *C. elegans* is intriguing. *Sir-2.2* and *sir-2.3* mutants have been shown to be less resistant to oxidative stress and have been shown to interact with mitochondrial biotin-dependent carboxylases involved in the TCA cycle (PYC-1), amino acid catabolism (PCAA-1), and formation of ketone bodies (MCCC-1).^27^ SIRT4, the mammalian orthologue of SIR-2.2/SIR-2.3, also interacts with these enzymes and malonyl CoA decarboxylase,^27^^,^^55^ suggesting that intermediates and/or outputs from these pathways may mediate mitochondria-to-HSF-1 signaling in fasted tissues. However, unlike our findings in worms, the levels of SIRT4 have been shown to decrease in muscle and liver tissue of fasted mice.^55^ In contrast, the levels of other mammalian mitochondrial sirtuins (SIRT3 and SIRT5) increase or remain unchanged in response to fasting in mouse tissue following fasting.^56^^,^^57^ Therefore, it is possible that different upstream processes couple mitochondria with HSF1 activity in mammals. Alternatively, the activity of mitochondrial sirtuins may increase in mammals during early fasting, before protein levels decline.

Lastly, our work reveals that the potentiation of HSF-1 activity, enhanced stress resistance and increased longevity are dependent on the H3K27me3 demethylase, JMJD-3.1. Increased JMJD-3.1 activity has been found to maintain the HSR in GSC deficient animals and promotes the UPR^mt^ under conditions of mitochondrial stress.^5^^,^^32^ JMJD-3.1 has also been shown to cooperate with the H3K27 acetyltransferase, CBP-1, to promote the UPR^mt^,^33^ and we also find that CBP-1 is necessary for fasting to fully enhance stress resistance. CBP-1 is also required for DR-mediated lifespan extension in worms,^58^ providing further support for the role of histone modifiers in promoting HSF-1 activity in the absence of food. However, CBP-1 has also been shown to acetylate and negatively regulate HSF-1 in worms and mammals under non-fasted conditions.^59^ This suggests that in the absence of food, CBP-1 switches its focus from regulating HSF-1 to acting on histones. Similarly, components of the nucleosome remodeling and deacetylase complex are required for both the HSR and UPR^mt^,^60^^,^^61^ and mitochondrial stress is associated with widespread chromatin reorganization via MET-2, LIN-65, and ISW-1.^47^^,^^62^ Therefore, future work to determine how fasting alters the acetylation status of HSF-1, and general chromatin organization, may shed further light on precisely how HSF-1 activity is potentiated at a select subset of PN genes during fasting.

We also observed that total histone H3 levels are elevated in fasted animals, suggesting that histone content, as well as status, are altered by fasting. Previous studies in flies and mice have demonstrated that inhibition of mTORC1 with rapamycin is also associated with elevated H3 and H4 levels.^63^^,^^64^^,^^65^ Therefore, increased histone levels and altered chromatin organization are likely a general phenomenon of metabolic remodeling. Given that overexpression of histones also promotes tissue health and longevity, it will be interesting to ascertain the extent to which these effects are mediated by potentiated HSF1 activity and enhanced proteostasis capacity.

In summary, the absence of food prior to reproductive maturity initiates a mitochondria-to-HSF-1 signaling axis through the activity of mitochondrial sirtuins and chromatin remodeling enzymes. This leads to an altered chromatin state that facilitates HSF-1 binding in response to protein folding stress, thereby protecting tissues against age-related proteostasis collapse. We propose that this mechanism exists to preserve tissue integrity in the face of additional external stresses during nutrient deprivation. In both *C. elegans* and mammals, this would provide greater organismal robustness when seeking new food sources in order to maximize fitness.

### Limitations of the study

While our work suggests a link between mitochondrial mass and HSF-1 activity, we do not formally demonstrate that changes in mitochondrial copy number in fasted animals are exclusively through clearance and not also due to reduced mitochondrial biogenesis. In addition, while increased SIR-2.2 levels are important for fasting to reduce mitochondrial copy number, we do not formally demonstrate that SIR-2.2 has a direct role in mitophagy. Lastly, while we demonstrate that both JMJD-3.1 and SIR-2.2 are necessary for transient fasting to increase lifespan, we have not resolved whether these factors interact with one another, or act through parallel pathways to augment longevity in response to temporary food removal.

## STAR★Methods

### Key resources table

**Table:** 

REAGENT or RESOURCE	SOURCE	IDENTIFIER
**Antibodies**		

Rabbit monoclonal anti Histone H3 - Nuclear Marker	ABCAM	Cat# ab176842; RRID:AB_2493104
Rabbit monoclonal anti Histone H3 (tri methyl K27)	ABCAM	Cat# ab192985; RRID:AB_2650559
Rabbit monoclonal anti Histone H3 (acetyl K27)	ABCAM	Cat# ab177178; RRID:AB_2828007
Mouse monoclonal anti-alpha tubulin	SIGMA	Cat# T5168; B512; RRID: AB_477579

**Bacterial and virus strains**		

*E. coli* OP50	CGC	WB OP50; RRID: WB-STRAIN:OP50
*E. coli* HT115	CGC	WB HT115; RRID: WB-STRAIN:HT115

**Chemicals, peptides, and recombinant proteins**		

Paraquat	SIGMA	Cat# 856177
Tunicamycin	Cambridge Bioscience	Cat# 11445

**Critical commercial assays**		

RNeasy extraction kit	QIAGEN	Cat# 74104
iScript cDNA synthesis kit	BIO-RAD	Cat# 1708890
Qiagen PCR purification kit	QIAGEN	Cat# 28104

**Deposited data**		

All raw and processed RNA-sequencing data	Gene Expression Omnibus	GEO: GSE236616

**Experimental models: Organisms/strains**		

*C. elegans*: STRAIN N2 Bristol: (wild type laboratory strain)	CGC	WB Strain: N2
*C. elegans*: STRAIN DA1116: *eat-2(ad1116)*	CGC	WB Strain: DA1116
*C. elegans*: STRAIN CF1903: *glp-1(e2144ts)*	CGC	WB Strain: CF1903
*C. elegans*: STRAIN MQ989: *isp-1(qm150);ctb-1(qm189)*	CGC	WB Strain: MQ989
*C. elegans*: STRAIN AM738: rmIs297 [*vha-6*p::Q(44)::YFP, *rol-6(su1006)*]	Gift from Morimoto Lab	WB Strain: AM738
*C. elegans*: STRAIN AM140: rmIs132 [*unc-54*p::Q(35)::YFP]	Gift from Morimoto Lab	WB Strain: AM140
*C. elegans*: STRAIN GMC101: dvIs100[unc-54p::Abeta-1-42::unc-54 3’ UTR + mtl-2p::GFP]	CGC	WB Strain: GMC101
*C. elegans*: STRAIN CL2122: dvIs15[pPD30.38) unc-54(vector) + (pCL26) mtl-2p::gfp]	CGC	WB Strain: CL2122
*C. elegans*: STRAIN PS3551: *hsf-1(sy441)*	CGC	WB Strain: PS3551
*C. elegans*: STRAIN CF1038: *daf-16(mu86)*	CGC	WB Strain: CF1038
*C. elegans*: STRAIN RB711: *pqm-1(ok485)*	CGC	WB Strain: RB711
*C. elegans*: STRAIN TM1153: *atf-6(tm1153)*	NBRP	WB Strain: TM1153
*C. elegans*: STRAIN SJ17: *xbp-1(zc12);hsp-4::GFP*	CGC	WB Strain: SJ17
*C. elegans*: STRAIN RB545: *pek-1(ok275)*	CGC	WB Strain: RB545
*C. elegans*: STRAIN TM4525: *atfs-1(tm4525)*	NBRP	WB Strain: TM4525
*C. elegans*: STRAIN JIN1375: *hlh-30(tm1978)*	CGC	WB Strain: JIN1375
*C. elegans*: STRAIN BR4006: *pink-1(tm1779)*	CGC	WB Strain: BR4006
*C. elegans*: STRAIN VC1024: *pdr-1(gk448)*	CGC	WB Strain: VC1024
*C. elegans*: STRAIN TM2648: *sir-2.2(tm2648)*	NBRP	WB Strain: TM2648
*C. elegans*: STRAIN RB654: *sir-2.3(ok444)*	CGC	WB Strain: RB654
*C. elegans*: STRAIN WBM321: (wbmIs321[acs-2p::gfp + rol-6(su1006)]),	Gift from Mair Lab	WB Strain: WBM321
*C. elegans*: STRAIN VC936: *jmjd-3.1(gk384)*	CGC	WB Strain: VC936
*C. elegans*: STRAIN SJ4103: *zcls14*[myo-3::GFP(mit)]	CGC	WB Strain: SJ4103
*C. elegans*: STRAIN TH188: ddIs105[sir-2.2::TY1::EGFP::3XFLAG(92C12) + unc-119(+)]	CGC	WB Strain: TH188
*C. elegans*: STRAIN MAH215: sqIs11[lgg-1p::mCherry::GFP::lgg-1 + rol-6(su1006)]	CGC	WB Strain: MAH215
*C. elegans*: STRAIN PP563: hhIs64[unc-119p(+); sur-5p::UbV::gfp]	Gift from Hoppe Lab	WB Strain: PP563
*C. elegans*: STRAIN PP556: hhIs57[unc-119(+); sur-5p::gfp]	Gift from Hoppe Lab	WB Strain: PP556
*C. elegans*: STRAIN STE68: *nhr-49(nr2041)*	CGC	WB Strain: STE68

**Oligonucleotides**		

Please see Table S3 for a complete list of probes used in the QuantiGene 16-plex assay	N/A	N/A
Please see Table S4 for a complete list of primers used in this study	N/A	N/A

**Software and algorithms**		

ImageJ	Schneider et al.^66^	https://imagej.nih.gov/ij/

### Resource availability

#### Lead contact

Further information and requests for resources and reagents should be directed to, and will be fulfilled by, John Labbadia: j.labbadia@ucl.ac.uk.

#### Materials availability

This study did not generate new, unique reagents.

#### Data and code availability

• RNA-seq data have been deposited at GEO and are publicly available as of the date of publication. Accession numbers are listed in the key resources table. All data contained in this paper will be shared by the lead author upon request.
• The paper does not report original code.
• Any additional information required to re-analyze the data reported in this paper is available from the lead contact upon request.

### Experimental model and study participant details

#### *C. elegans* strains, culture conditions and fasting

All strains were maintained at 20°C on NGM plates seeded with *Escherichia coli* OP50 using standard husbandry techniques.^67^ Strains used in this study were: N2 Bristol (wild type laboratory strain), AD1116 *eat-2(ad1116)*, CF1903 *glp-1(e2144ts)*, MQ989 *isp-1(qm150);ctb-1(qm189)*, AM738 (rmIs297 [*vha-6*p::Q(44)::YFP, *rol-6(su1006)*]), AM140 (rmIs132 [*unc-54*p::Q(35)::YFP]), GMC101 dvIs100[unc-54p::Abeta-1-42::unc-54 3’ UTR + mtl-2p::GFP], CL2122 dvIs15[pPD30.38) unc-54(vector) + (pCL26) mtl-2p::gfp], PS3551 *hsf-1(sy441),* CF1038 *daf-16(mu86),* RB711 *pqm-1(ok485)*, TM1153 *atf-6(tm1153)*, SJ17 *xbp-1(zc12)*, RB545 *pek-1(ok275)*, TM4525 *atfs-1(tm4525)*, JIN1375 *hlh-30(tm1978)*, BR4006 *pink-1(tm1779)*, VC1024 *pdr-1(gk448)*, TM2648 *sir-2.2(tm2648)*, RB654 *sir-2.3(ok444)*, WBM321 (wbmIs321[acs-2p::gfp + rol-6(su1006)]), STE68 *nhr-49(nr2041)*, VC936 *jmjd-3.1(gk384)*. SJ4103 *zcls14*[myo-3::GFP(mit)], TH188 ddIs105[sir-2.2::TY1::EGFP::3XFLAG(92C12) + unc-119(+)], MAH215 sqIs11[lgg-1p::mCherry::GFP::lgg-1 + rol-6(su1006)], PP563 hhIs64[unc-119p(+); sur-5p::UbV::gfp] and PP556 hhIs57[unc-119(+); sur-5p::gfp]. Worms were fasted by moving mid-stage L4s to plates without food for indicated lengths of time (4, 8, 12, 16, 20, or 24 hours). Following fasting, worms were moved back to plates containing food for subsequent stress resistance or lifespan assays. To ensure efficient removal of food from roller strains (those carrying a *rol-6* selection marker), mid-L4 stage worms were sequentially moved to plates without food three times, with 10-minute periods of incubation/crawling allowed between each transfer.

#### Mouse breeding and maintenance

All procedures were performed in accordance with the Animals (Scientific Procedures) Act 1986, complied with ARRIVE guidelines and were approved by the University College London Ethical Review Process Committee. Animals used for fasting and QuantiGene analysis experiments were (CBA/Ca × C57Bl/6J)F1 mice (B6CBAF1/OlaHsd, Envigo). Male mice at 11 weeks of age were used for all treatment groups. Mouse husbandry was performed with up to five mice housed per cage. Mice were housed in individually ventilated cages with Aspen Chips 4 Premium bedding (Datesand) and with environmental enrichment which included chew sticks and a play tunnel (Datesand). Mice had unrestricted access to food (Teklad global 18% protein diet, Envigo) and water. The temperature was regulated at 21°C ± 1°C and animals were kept on a 12 h light/dark cycle. The animal facility was barrier-maintained and quarterly non-sacrificial FELASA screens found no evidence of pathogens.

#### NVP-HSP990 formulation and dosing of fed and fasted mice

NVP-HSP990 (2-amino-7,8-dihydro-6H-pyrido[4,3-d]pyrimidin-5-one) was obtained from Novartis Pharma AG. NVP-HSP990 was formulated as a suspension in 2% methylcellulose (Sigma), diluted in 0.9% saline solution (Severn Biotech) and sonicated twice at high frequency in an ultrasonic bath. Both vehicle and NVP-HSP990 solutions were freshly prepared for the dosing experiment. Thorough mixing was carried out between doses to maintain NVP-HSP990 as an even suspension. Male mice at 11 weeks of age were fasted by complete removal of food for 16 hours. At this point, animals were dosed with vehicle (n = 6 fed; n = 5 fasted) or 12 mg / kg NVP-HSP990 (n = 9 fed; n = 9 fasted) by oral gavage and sacrificed 2 hours later (total of 18 hours without food for fasted group). Sample sizes were based on previous experimental results.^34^ For this dosing experiment, male wild-type mice were always randomized with respect to litter of origin and age matched at 11 weeks of age. 2 hours after treatment with NVP-HSP990 or vehicle, mice were sacrificed by a schedule 1 procedure, dissected, tissues were snap-frozen in liquid nitrogen and stored at − 80°C.

### Method details

#### Tissue homogenization and QuantiGene gene expression assays

QuantiGene experiments were performed as previously described.^34^ Tissue samples were homogenised using a Polytron homogeniser for brain regions or liquid nitrogen and pre-chilled pestle and mortar for muscle, using QuantiGene reagents from Thermo Fisher Scientific, following the manufacturer’s recommendations. Housekeeping genes used for normalization in cerebrum (brain) tissue were *Canx, Atp5b, Eif4a2, Sdha, Gapdh* and *Rpl13a*. For quadricep (muscle) tissue, *Canx, Atp5b, Eif4a2,* and *Sdha* were used. The tissue lysate dilutions used for this study are listed in Table S2. Information pertaining to probe regions and accession numbers for the QuantiGene multiplex assays used in this study can be found in Table S3. The median fluorescent intensity (MFI) was read in a Magpix (Luminex) using the xPonent software.

#### RNA interference

All clones were sequenced verified before use and were obtained from the Ahringer RNAi library. RNAi was performed by growing bacteria for 16 hours at 37°C in LB containing 100 μg/ml ampicillin, with shaking (220 rpm). Cultures were then induced with 5 mM IPTG and allowed to grow at 37°C for a further 3 hours. Following induction, bacteria were allowed to cool at room temperature and were then seeded onto NGM plates containing 100 μg/ml ampicillin and 1 mM IPTG. Seeded plates were allowed to dry at room temperature before use.

#### Lifespan and stress resistance assays

In all lifespan and stress resistance assays, survival was scored by gently touching worms with a platinum pick at indicated time points. Worms were scored as dead in the absence of touch response and absence of pharyngeal pumping. In lifespan assays, worms exhibiting intestinal prolapse through the vulva (rupturing) or internal hatching of progeny (bagging) were censored. For thermorecovery assays, worms were heat shocked on seeded NGM plates at 35°C for 4 hours and allowed to recover at 20°C. For tunicamycin or paraquat treatment, worms were transferred to seeded NGM plates containing tunicamycin (50 μg/ml) or paraquat (10 mM) and maintained at 20°C until dead.

#### Proteostasis sensor assays

Polyglutamine aggregation was scored in muscle and intestinal proteostasis sensors at indicated time points under a Nikon SMZ1270 fluorescence dissecting stereomicroscope. Aggregates were determined to be any discrete foci exhibiting fluorescence signal above the background diffuse signal. Muscle function was assessed in polyglutamine expressing animals by scoring paralysis at different days of adulthood. Animals were scored as paralysed when they were unable to move forwards or backwards at least one body length in response to touch with a platinum pick. For Abeta (GMC101) and control (CL2122) animals, muscle health was assessed by measuring thrashing ability in M9 buffer (number of body bends per minute) under a Leica M80 stereo dissection microscope. Worms were shifted to 25 °C at the L4 stage to induce paralysis/thrashing defects. All scoring was performed blind to treatment groups.

#### Autophagy reporter

Animals were synchronised by egg lay for 1 hour at 20°C. Once animals reached the L4 stage, they were transferred onto NGM plates with or without food for 24 hours. After this period, worms were moved back to plates containing food for 24 hours, at which point, fed and fasted animals were heat-shocked in a water bath for 4 hours at 35°C, or kept at 20°C for an equivalent period of time. Immediately following heat shock, worms were mounted onto slides for image acquisition. Worms were mounted on 2% agarose pads in 5 mM levamisole and imaged immediately. For image acquisition, a Zeiss Imager.Z2 microscope with a Hamamatsu C13440 ORCA-Flash4.0 V3 digital camera, Apotome.2 for Z-stack images and ZenBlue software were used. Z-stacks were acquired at 0.6 μm slice intervals using 100x objective and processed as maximum intensity projections. Based on previous studies,^68^ a Z-position was selected where the nucleus could be seen in the intestine and posterior pharyngeal bulb in the pharynx. The area of the first two intestinal cells was used for the quantification of visible puncta in the intestine, whereas the area of the posterior pharyngeal bulb was used for the quantification of visible puncta in the pharynx. Green or yellow puncta were scored as autophagosomes while red puncta were scored as autolysosomes.

#### UPS reporter

Animals were synchronised by egg lay for 1 hour at 20°C. Once animals reached the L4 stage, they were transferred onto NGM plates with or without food for 24 hours. After this period, worms were moved back to plates containing food for 24 hours, at which point, fed and fasted animals were heat-shocked in a water bath for 4 hours at 35°C, or kept at 20°C for an equivalent period of time. Immediately following heat shock, worms were mounted onto slides for image acquisition. Worms were mounted on 2% agarose pads in 3 mM levamisole and imaged immediately. For image acquisition, a Nikon SMZ1270 fluorescence dissecting stereomicroscope with a DS-Fi3 5.9 MP colour camera was used. Images were processed using ImageJ.^66^

#### RNA extraction, cDNA synthesis and RTqPCR

Approximately 100-200 adult animals per treatment group were lysed in 250 μl of Trizol by vortexing for 20 minutes at 4°C. RNA was purified using an RNeasy extraction kit as per manufacturer’s instructions. cDNA was generated using 1 μg of total RNA and an iScript cDNA synthesis kit. Real-time quantitative PCR was performed using a Biorad CFX96 Real-time PCR detection system and BioRad SsoAdvanced SYBR green super mix. Expression of genes of interest was calculated relative to the housekeeping genes *rpb-2* and *cdc-42* using the standard curve method. Sequences for all primer pairs used in this study can be found in Table S3. For quantification of mRNA levels following heat shock, animals were harvested immediately after the heat shock conditions specified (35°C, 30 minutes).

#### RNA-sequencing and analysis

RNA integrity was assessed using an RNA Nano 6000 assay kit and an Agilent Bioanalyzer 2100 system. Following this, mRNA was purified from 1 μg of total RNA using poly-dT magnetic beads and cDNA libraries were generated using random hexamers and M-MuLV reverse transcriptase for first strand synthesis, followed by second strand synthesis using DNA polymerase I and RNase H. Fragments were blunt-ended and adapters were ligated before PCR was performed using Phusion high fidelity DNA polymerase. PCR products were purified using an AMPure XP system and library quality was checked using an Agilent Bioanalyzer 2100. Libraries were sequenced using an Illumina Novaseq 6000 platform to generate paired-end 150 base pair reads at a depth of 20 million reads per sample. Following sequencing, raw data were checked and reads containing adapter sequences, poly-N reads or poor-quality sequences were removed. Reads were then aligned to the *C. elegans* reference genome using Hisat2 v2.0.5 and featureCounts v1.5.0-p3 was used to quantify the number of reads mapped to each gene. Differential expression testing was carried out using DESeq2 and p-values were adjusted using Benjamini-Hochberg. Z-scores were calculated for each gene from log FPKM expression data for all differentially expressed genes (> 1.5-fold, adj p < 0.05). Up or down regulated genes were analysed using the g:profiler tool to identify KEGG processes/pathways and Gene Ontology categories that were enriched in either group. Raw and processed data can be found at the NCBI Gene Expression Omnibus using accession number GEO: GSE236616. For quantification of mRNA levels following heat shock, animals were harvested immediately after the heat shock conditions specified (35°C, 30 minutes).

#### Protein extraction and western blotting

To extract protein for western blotting, worms (approximately 500 – 1000) were collected in M9, pelleted, and then resuspended in RIPA buffer supplemented with a protease inhibitor cocktail tablet. Worm pellets were then flash frozen in liquid nitrogen and ground in microcentrifuge tubes using a plastic dounce homogenizer. Freezing and grinding were performed twice, and effectiveness of lysing was confirmed by checking a sample of the lysate under a dissecting microscope. Lysates were then centrifuged at 15,000 × *g* at 4°C for 15 minutes and the supernatant was collected. Proteins were separated by SDS-PAGE and transferred to nitrocellulose membranes before probing with primary antibodies. Blots were incubated with primary antibodies for 1 hour at room temperature (Histone H3 - 1:5000, tubulin - 1:10,000) or overnight at 4°C (anti-H3K27me3 (1:1000), anti-H3K27ac (1:1000)), washed three times with PBS-0.2% Tween, incubated with secondary antibodies for 1 hour at room temperature (mouse-HRP – 1:5000, rabbit-HRP – 1:5000), washed a further three times with PBS-0.2% Tween, and then developed using ECL detection reagents and an Amersham ImageQuant800 detection system. Densitometry of protein bands was performed using ImageJ gel analysis tools.

#### Chromatin immunoprecipitation

Worms (20,000 per treatment group) were harvested in M9, pelleted and re-suspended in 1% formaldehyde-PBS to promote cross-linking. Worms were then fixed for 30 minutes at room temperature, washed three times in PBS and resuspended in FA buffer. Worms were then dounce homogenized on ice before being subjected to sonication using a Diagenode Bioruptor sonicator (15 rounds of 30 s on 1 min off). Samples were then centrifuged at 4°C for 15 minutes and lysates were subjected to 5 more rounds of sonication to shear chromatin to approximately 500 bp in size. Pulldowns were performed by incubating 2 mg of pre-cleared chromatin with 20 μl of washed and pre-blocked Protein G Dynabeads and 2 μg antibody (anti-GFP) in 1 ml of FA buffer, overnight at 4°C. Following incubation, beads were washed twice with FA buffer, once with low salt wash buffer, once with high salt wash buffer, and once with TEL buffer before being eluted from beads using 100 μl of 1% SDS. Cross-linking was reversed by incubating samples at 65°C overnight in the presence of 20 mM NaCl, before protein and RNA were removed by RNAse A (30 min at 37°C) and Proteinase K (1 hour at 55°C) treatment. Samples were boiled at 95°C and DNA was purified using a Qiagen PCR purification kit, as per manufacturer’s instructions.

#### Mitochondrial morphology assays

The mitochondria morphology in muscle of the *zcls14[myo-3::GFP(mit)]* animals was examined. 2% of molten agarose in water was used to make glass slides. Animals were immobilized on agarose pads in 3 mM levamisole with a glass cover slip on the top. To acquire images, 63x/1.40 oil objective lens of the Zeiss Imager.Z2 microscope was used. Images were processed using ImageJ. Qualitative analysis of the mitochondria morphology in each muscle section was performed. The largest regions of clear body wall muscle were identified between the pharynx and the vulva or between the vulva and tail. No significant difference was observed between these two segments. Adjacent regions to the tail, pharynx and vulva were excluded due to the disrupted mitochondria morphology which naturally occurs there. Images were scored as fused, tubular, intermediate and fragmented based on the mitochondria structure organization.

### Quantification and statistical analysis

All statistical tests (Mantel Cox Log-rank, one-way ANOVA, two-way ANOVA and Student’s t-test) were carried out as stated within each figure legend using GraphPad Prism 9. The statistical details of all experiments can be found within the accompanying figure legends or in Table S1.

## Supplemental information

Document S1. Figures S1–S6 and Tables S2–S4

Table S1. Statistics for all lifespan and stress resistance assays, related to Figures 1, S1, 2, S2, 3, 4, S4, 5, and S5
