# Supplementary material for: Mitochondrial clearance and increased HSF-1 activity are coupled to promote longevity in fasted Caenorhabditis elegans
Source: iScience. 2024 Apr 27;27(6):109834. doi: 10.1016/j.isci.2024.109834 (PMC11112483; doi:10.1016/j.isci.2024.109834)
Supplement: Document S1. Figures S1–S6 and Tables S2–S4 [file mmc1.pdf]

iScience, Volume *27*

Supplemental information

Mitochondrial clearance and increased HSF-1 activity are coupled to promote longevity in fasted *Caenorhabditis elegans*

Nikolaos Tataridas-Pallas, Yahyah Aman, Rhianna Williams, Hannah Chapman, Kevin J.H. Cheng, Casandra Gomez-Paredes, Gillian P. Bates, and John Labbadia

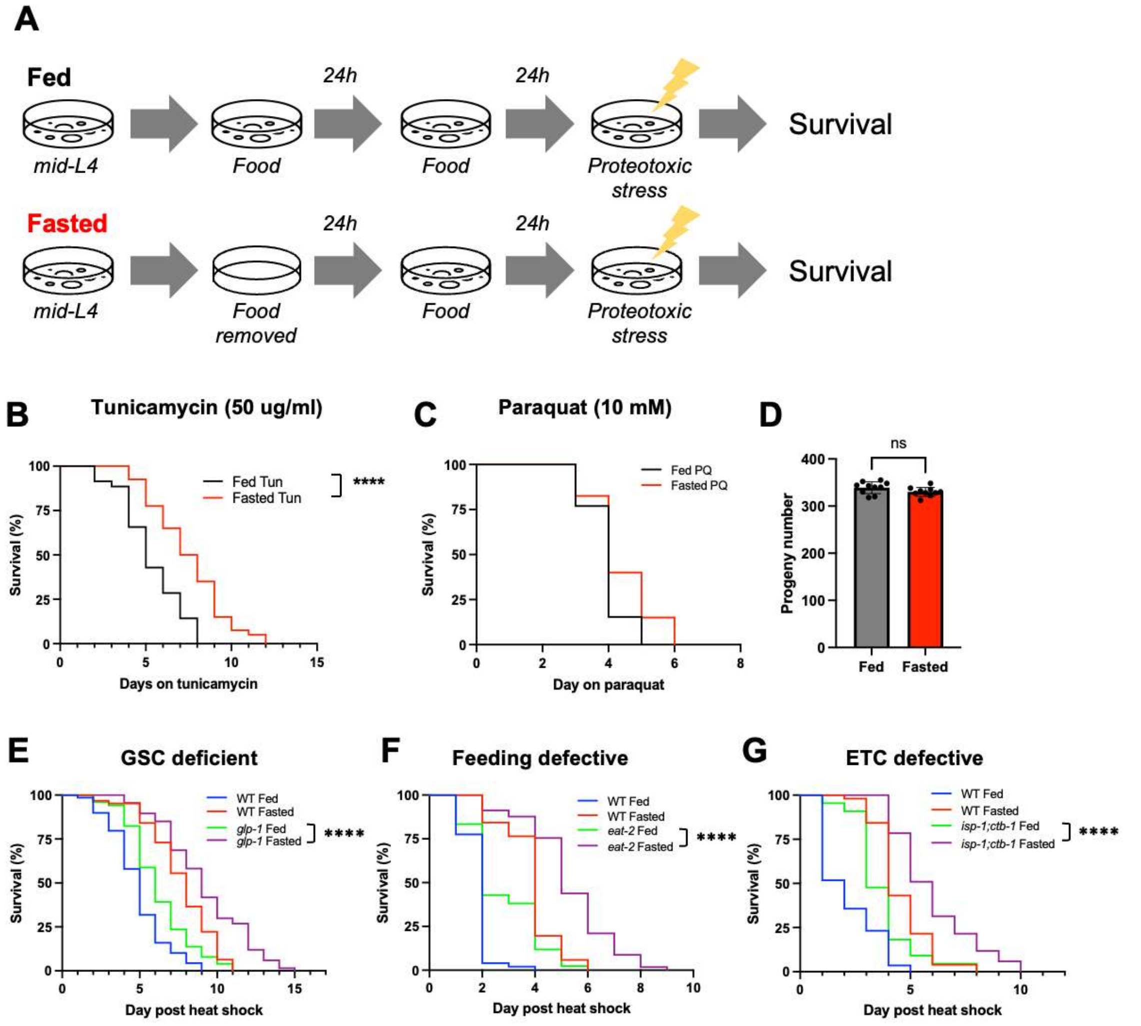

Supplemental Figure 1: Transient fasting at the transition to adulthood does not reduce total progeny number (related to Figure 1)

(A) Schematic of fasting regimen employed. **(B & C)** Survival of fed and fasted worms following **(B)** tunicamycin or **(C)** paraquat treatment. **(D)** Total number of progeny produced overall in fed and fasted animals. Data are plotted as mean +/- SD. **(E - G)** Survival of fed and fasted wild-type (N2), **(E)** germline stem cell (GSC) deficient (*glp-1(e2144ts)*), **(F)** feeding defective (*eat-2(ad1116)*) and **(G)** electron transport chain (ETC) impaired (*isp-1(qm150);ctb-1(q89)*) worms following heat shock (HS) (35°C, 4 hours). Statistical comparisons were made using Mantel-Cox Log Rank test (B) and Student's unpaired t-test (D). ns = $p > 0.05$, **** = $p < 0.0001$. Full statistics for survival curves can be found in Supplemental Table 1. Fasting conditions were as follows: B-D - animals were removed from food for 24 hours starting at the L4 stage and then returned to food thereafter; E-G - animals were removed from food for 24 hours starting at the L4 stage and then returned to food for 24 hours prior to HS;

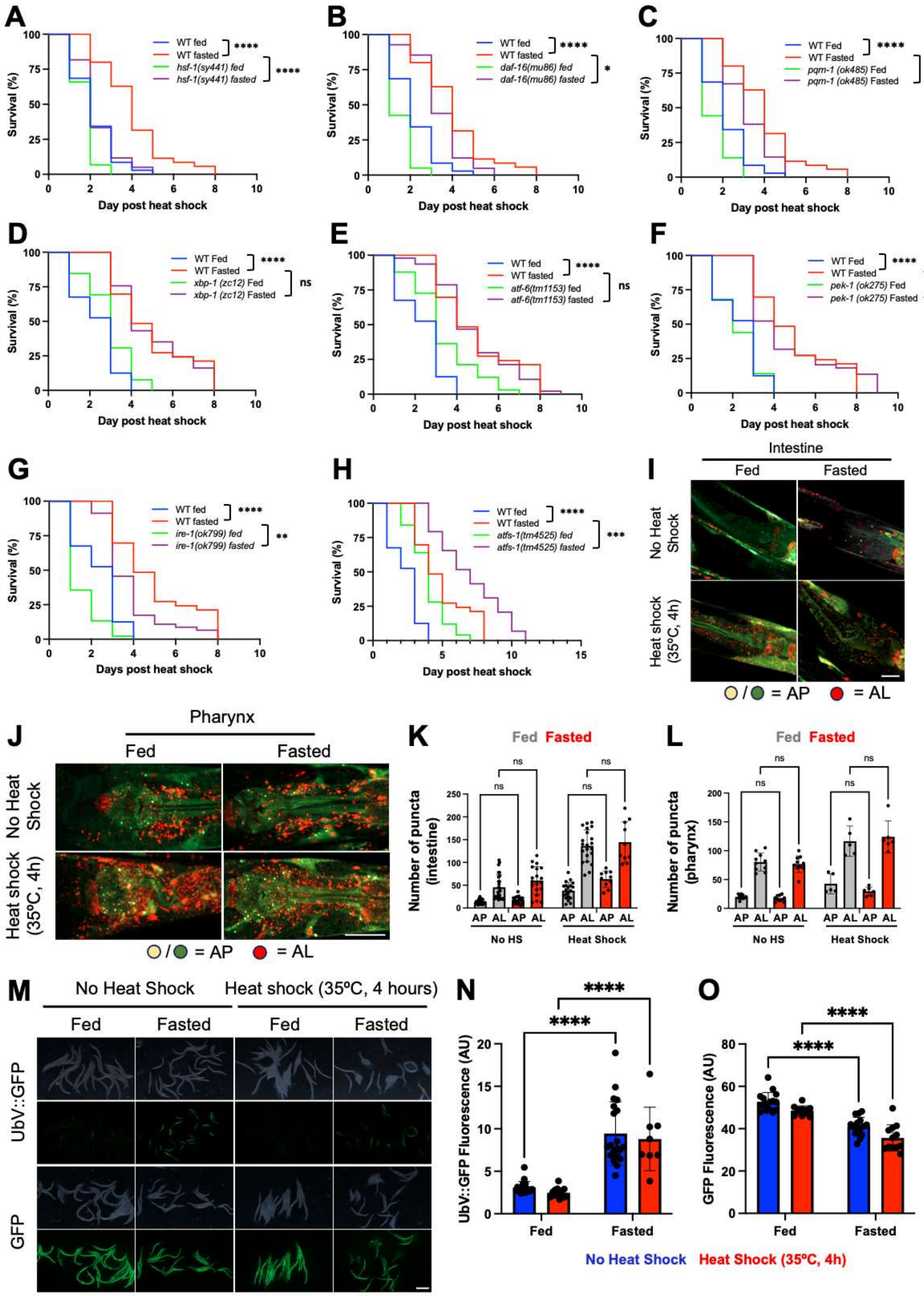

A
B
C
D
E
F
G
H
I
J
K
L
M
N
O
WT fed
WT fasted
hsf-1(sy441) fed
hsf-1(sy441) fasted
daf-16(mu86) fed
daf-16(mu86) fasted
pqm-1 (ok485) Fed
pqm-1 (ok485) Fasted
xbp-1 (zc12) Fed
xbp-1 (zc12) Fasted
atf-6(tm1153) fed
atf-6(tm1153) fasted
pek-1 (ok275) Fed
pek-1 (ok275) Fasted
ire-1(ok799) fed
ire-1(ok799) fasted
atfs-1(tm4525) fed
atfs-1(tm4525) fasted
Survival (%)
Day post heat shock
Days post heat shock
Intestine
Pharynx
Fed
Fasted
No Heat Shock
Heat shock (35°C, 4h)
= AP
= AL
Number of puncta (intestine)
Number of puncta (pharynx)
No HS
Heat Shock
Heat shock (35ºC, 4 hours)
UbV::GFP
GFP
UbV::GFP Fluorescence (AU)
GFP Fluorescence (AU)
No Heat Shock
Heat Shock (35ºC, 4h)

Supplemental Figure 2: Fasting does not enhance proteostasis capacity through the UPR^{ER} or UPR^{mt} (related to Figure 2)

(A - H) Survival of fed and fasted wild-type (N2) or PN mutant animals following heat shock (35°C, 4 hours). **(I & J)** Representative images of the **(I)** intestine or **(J)** pharynx of fed or fasted worms expressing GFP::LGG-1::mCHERRY and exposed to basal conditions (20°C) or heat shock (35°C) for 4 hours. Yellow and green puncta represent autophagosomes (AP), red puncta represent autolysosomes (AL). Scale bars = 20μM **(K & L)** Number of AP or AL in the **(K)** intestine or **(L)** pharynx of fed or fasted worms expressing GFP::LGG-1::mCHERRY and exposed to basal conditions (20°C) or heat shock (35°C) for 4 hours. Worms were imaged immediately following heat shock. **(M)** Representative images of fed and fasted worms expressing UbV::GFP or GFP alone following exposure to basal conditions (20°C) or heat shock (35°C) for 4 hours. Worms were imaged immediately following heat shock. Scale bar = 500μM. **(N & O)** Fluorescence intensity of **(N)** UbV::GFP or **(O)** GFP alone in fed and fasted worms following exposure to control conditions or heat shock (35°C, 4h). Data are plotted as mean +/- SD. Statistical comparisons were made using Mantel-Cox Log-Rank test (A-H) or Two-way ANOVA with pairwise comparison of groups (K, L, N and O). ns = $p > 0.05$, * = $p < 0.05$, ** = $p < 0.01$, *** = $p < 0.001$, **** = $p < 0.0001$. Full statistics for survival curves can be found in Supplemental Table 1. Survival assays in A-C and D-H were run in parallel. Fasting conditions were as follows: A-O - animals were removed from food for 24 hours starting at the L4 stage and then returned to food for 24 hours prior to HS or imaging.

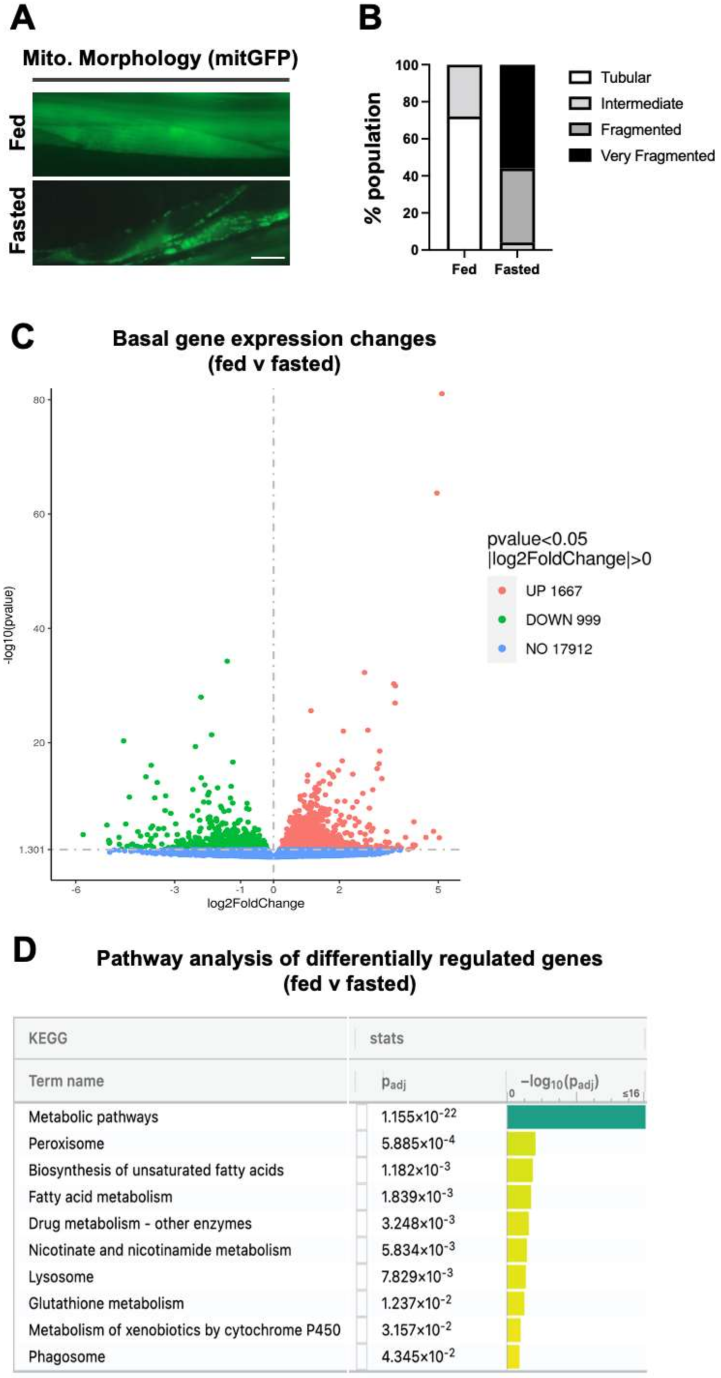

Supplemental Figure 3: Early life fasting induces mitochondrial and metabolic remodelling (related to Figure 3)

(A) Representative images of mitochondrial morphology (GFPmt) in fed and fasted animals. Scale bar = 10μM. **(B)** Relative incidence of Tubular, Intermediate, Fragmented and Very fragmented mitochondria in fed and fasted worms. **(C)** Volcano plot of up-regulated and down-regulated genes in fasted worms under basal conditions. **(D)** KEGG pathway analysis of differentially expressed genes in fasted worms. Fasting conditions were as follows: A-D - animals were removed from food for 24 hours starting at the L4 stage and then immediately imaged or collected.

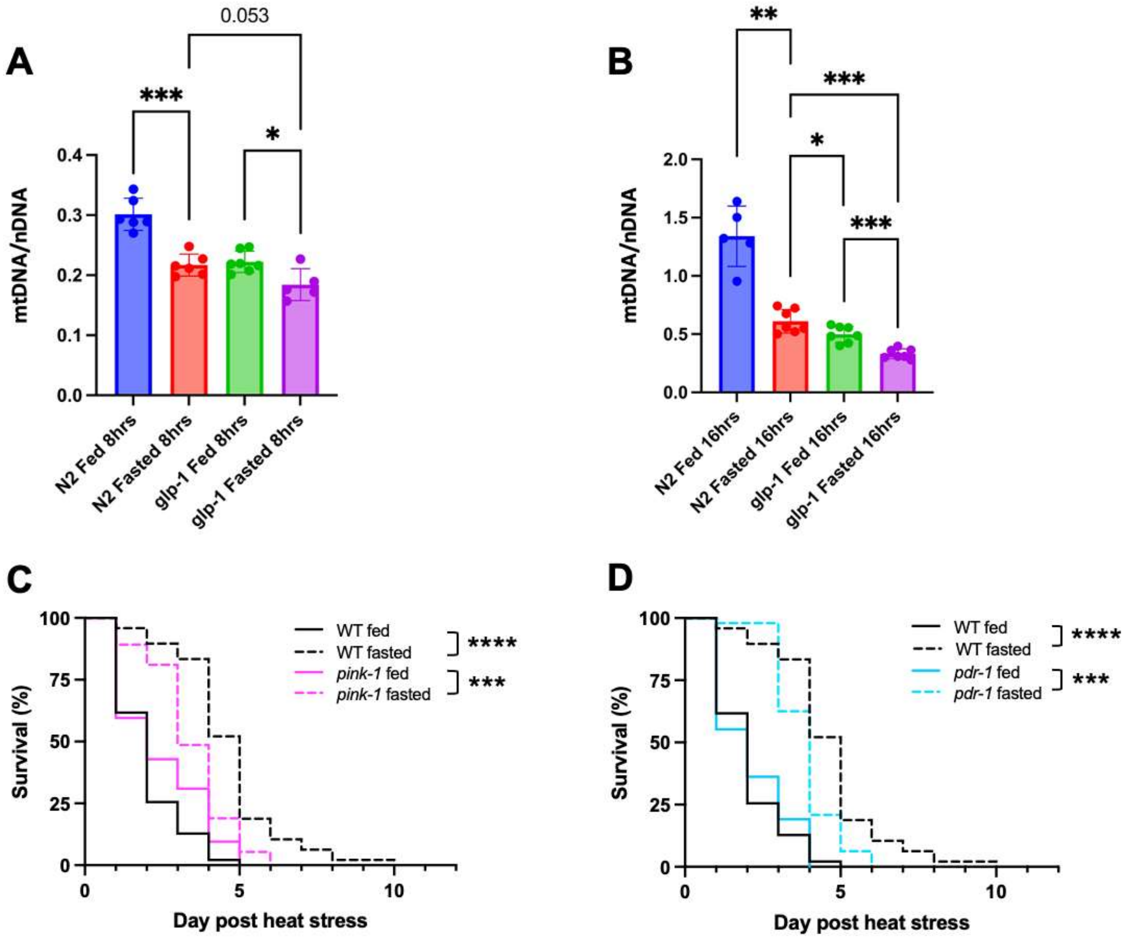

Supplemental Figure 4: Defective mitophagy suppresses stress resistance in fasted worms (related to Figure 4)

(A & B) Relative mitochondrial copy number in fed and fasted wild-type worms and germline stem cell (GSC) deficient (*glp-1(e2144ts)*) mutants at **(A)** 8 hours **or (B)** 16 hours post fasting. Data are plotted as mean +/- SD. **(C & D)** Survival of fed and fasted wild-type (N2), **(C)** *pink-1* and **(D)** *pdr-1* mutant worms following heat shock (HS) (35°C, 4 hours). Statistical comparisons were made using two-way ANOVA with post-analysis pairwise comparison of groups (A & B) and Mantel-Cox Log Rank test (C & D). * = $p < 0.05$, ** = $p < 0.01$, *** = $p < 0.001$, **** = $p < 0.0001$. Full statistics for survival curves can be found in Supplemental Table 1. Survival assays in C and D were run in parallel with Figure 4J. Fasting conditions were as follows: A & B - animals were removed from food for 24 hours starting at the L4 stage for the indicated times and then immediately collected; C & D - animals were removed from food for 24 hours starting at the L4 stage and then returned to food for 24 hours prior to HS.

.

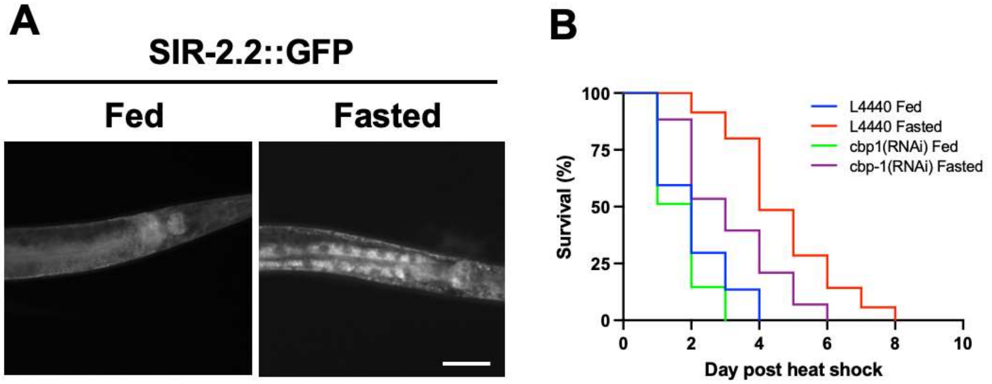

Supplemental Figure 5: CBP-1 is required for the maintenance of proteostasis in fasted animals (related to Figure 5)

(A) Representative images of fed and fasted SIR-2.2::EGFP worms. Scale bar = 100μM. **(B)** Survival of fed and fasted L4440 and *cbp-1*(RNAi) treated worms following heat shock (35°C, 4 hours). Full statistics for survival curves can be found in Supplemental Table 1. Fasting conditions were as follows: A - animals were removed from food for 24 hours starting at the L4 stage and then immediately imaged; B - animals were removed from food for 24 hours starting at the L4 stage and then returned to food for 24 hours prior to HS.

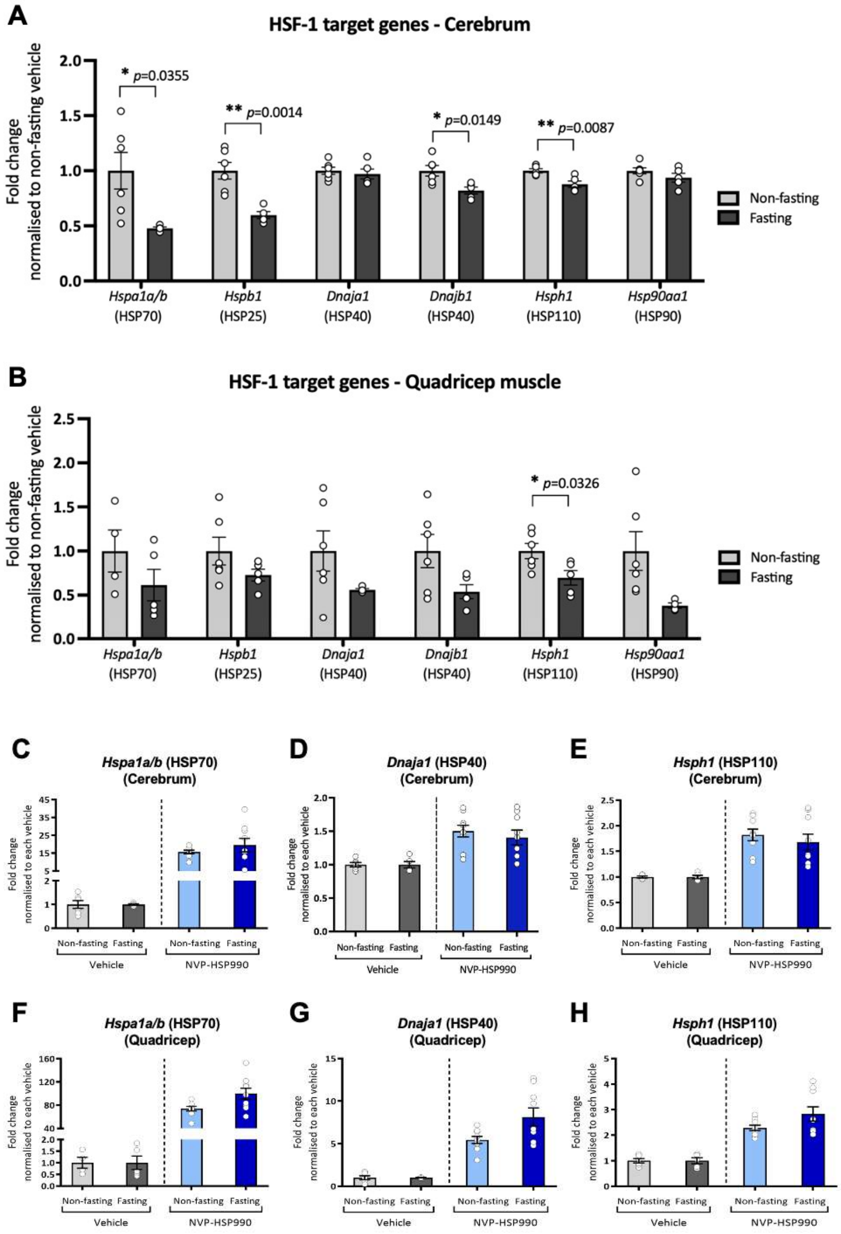

Supplemental Figure 6: Fasting does not increase the expression of HSF-1 target genes in vehicle-treated mice (related to Figure 6)

(A & B) QuantiGene assessment of HSF-1 target genes in **(A)** brain tissue (cerebrum) and **(B)** quadricep muscle of fed and fasted vehicle-treated mice. **(C - E)** QuantiGene assessment of HSF-1 target genes in brain tissue (cerebrum) of fed and fasted mice following treatment with vehicle or NVP-HSP990 (12 mg/kg). **(F – H)** QuantiGene assessment of HSF-1 target genes in quadricep muscle tissue of fed and fasted mice following treatment with vehicle or NVP-HSP990 (12 mg/kg). Data are plotted as mean +/- SEM. Statistical comparisons were made using unpaired Student's t-test (A & B) or two-way ANOVA with Bonferroni correction (C-H). Data were screened for outliers using a ROUT test and one mouse was removed from both the NVP-HSP990 fed and fasted groups. Final sample numbers were: vehicle non-fasting = 6, vehicle fasted = 5, NVP-HSP990 non-fasting = 9, NVP-HSP990 fasted = 9.

Supplemental Table 2: Tissue dilutions used in the QuantiGene 16-plex assay (related to Figure 6, Figure S6 and STAR methods)

Tissue	Dilution of starting material (10 mg/ 300 uL)	Final input (ug/ul)
**Brain (cerebrum)**	1:9	3.7
**Quadricep muscle**	1:2	16.6

Supplemental Table 3: Probes in the QuantiGene 16-plex assay (Related to Figure 6, Figure S6 and STAR methods)

Type	Gene Symbol	Gene name	Accession Number	Probe set region
HK	*Canx*	*Calnexin*	*NM_007597*	*1195-1720*
HK	*Rpl13a*	*Ribosomal Protein L13a*	*NM_009438*	*2-467*
HK	*Atp5b*	*ATP synthase subunit Beta*	*NM_016774*	*22-409*
HK	*Eif4a2*	*Eukaryotic translation initiation factor 4A2*	*NM_013506*	*710-1271*
HK	*Sdha*	*Succinate dehydrogenase complexflavoprotein subunit A*	*NM_023281*	*76-727*
HK	*Gapdh*	*Glyceraldehyde 3-phosphate dehydrogenase*	*NM_001001303*	*735-1001*
GOI	*Hsf1*	*Heat shock factor 1*	*NM_008296*	*1712-2263*
GOI	*Hspa1a/b*	*Heat shock protein 1A/B*	*NM_010479*	*2186-2721*
GOI	*Hspb1*	*Heat shock protein 1*	*NM_013560*	*103-555*
GOI	*Dnaja1*	*DnaJ (Hsp40) homolog, subfamilyA, member 1*	*NM_008298*	*474-1101*
GOI	*Dnajb1*	*DnaJ (Hsp40) homolog, subfamily Bmember 1*	*NM_018808*	*569-1125*
GOI	*Hspd1*	*Heat shock protein 1 (chaperonin)*	*NM_010477*	*1732-2170*
GOI	*Hspe1*	*Heat shock protein 1 (chaperonin10)*	*NM_008303*	*259-657*
GOI	*Hsph1*	*Heat shock protein 105*	*NM_013559*	*1985-2430*
GOI	*Hsp90aa1*	*Heat shock protein 90, alpha(cytosolic), class A member 1*	*NM_010480*	*245-989*
GOI	*Hsp90ab1*	*Heat shock protein 90, alpha(cytosolic), class B member 1*	*NM_008302*	*1039-1542*

GOI = Gene of interest; HK = Housekeeping gene

Supplemental Table 4: Primers used in this study (related to Figures 2, 4 and 5)

Target	Forward	Reverse
rpb-2	AACTGGTATTGTGGATCAGGTG	TTTGACCGTGTCGAGATGC
pmp-3	GTTCCCGTGTTCATCACTCAT	ACACCGTCGAGAAGCTGTAGA
cdc-42	TCGACAATTACGCCGTCACA	GAAACACGTCGGTCTGTGGA
hsp-6	AACCATTGAGCCATGCCGTA	CTTGAACAGTGGCTTGCACC
hsp-4	GGGGACAATCATTGGTATCG	ACGCAACGTATGATGGAGTG
hsp-16.2	AGATGTAGATGTTGGTGCAGT	TCTCTTCGACGATTGCCTGT
hsp-70 (C12C8.1)	CTACATGCAAAGCGATTGGA	GGCGTAGTCTTGTTCCCTTC
hsp-70b (F44E5.4/5)	TGATACCCATCTCGGAGGAG	GTGGATTGGGTGAAATGTCC
sir-2.2	TTAGCCTGGCCGAGATTTGG	AGTGAAGCCCATCCACGTTT
sir-2.3	GGGTAGCGAACAAGGGTTCA	GGTTCTCCCCAAACAACGTTAC
hsp-16.2 promoter	TCCATGTACCGAATGTGAGTC	TGTTCGGTATTTATTTTCAACGGT
hsp-70 promoter	ATAGCATAGGCGACCCACAG	ACGTTCCTCTGGCATCTTCT
hsp-70b promoter	CCAGCTGCATCACTCTGTCT	GGCCGACAGAAGAGACAACA
cdc-42 promoter	GTAAAGAAACGCTCGTGGCA	GATCGTCTGCATTTCGCCTG
